# Identification of microbial markers across populations in early detection of colorectal cancer

**DOI:** 10.1038/s41467-021-23265-y

**Published:** 2021-05-24

**Authors:** Yuanqi Wu, Na Jiao, Ruixin Zhu, Yida Zhang, Dingfeng Wu, An-Jun Wang, Sa Fang, Liwen Tao, Yichen Li, Sijing Cheng, Xiaosheng He, Ping Lan, Chuan Tian, Ning-Ning Liu, Lixin Zhu

**Affiliations:** 1 Department of Gastroenterology, The Shanghai Tenth People’s Hospital, Department of Bioinformatics, School of Life Sciences and Technology, Tongji University, Shanghai, People’s Republic of China; 2 Guangdong Institute of Gastroenterology, Guangdong Provincial Key Laboratory of Colorectal and Pelvic Floor Diseases, Department of Colorectal Surgery, The Sixth Affiliated Hospital, Sun Yat-sen University, Guangzhou, People’s Republic of China; 3 Department of Biomedical Informatics, Harvard Medical School, Boston, MA USA; 4 State Key Laboratory of Oncogenes and Related Genes, Center for Single-Cell Omics, School of Public Health, Shanghai Jiao Tong University School of Medicine, Shanghai, People’s Republic of China; 5 School of Medicine, Sun Yat-sen University, Shenzhen, People’s Republic of China; 6 Genome, Environment and Microbiome Community of Excellence, The State University of New York at Buffalo, Buffalo, NY USA

**Keywords:** Clinical microbiology, Diagnostic markers, Colorectal cancer

## Abstract

Associations between gut microbiota and colorectal cancer (CRC) have been widely investigated. However, the replicable markers for early-stage adenoma diagnosis across multiple populations remain elusive. Here, we perform an integrated analysis on 1056 public fecal samples, to identify adenoma-associated microbial markers for early detection of CRC. After adjusting for potential confounders, Random Forest classifiers are constructed with 11 markers to discriminate adenoma from control (area under the ROC curve (AUC) = 0.80), and 26 markers to discriminate adenoma from CRC (AUC = 0.89), respectively. Moreover, we validate the classifiers in two independent cohorts achieving AUCs of 0.78 and 0.84, respectively. Functional analysis reveals that the altered microbiome is characterized with increased ADP-l-glycero-beta-d-manno-heptose biosynthesis in adenoma and elevated menaquinone-10 biosynthesis in CRC. These findings are validated in a newly-collected cohort of 43 samples using quantitative real-time PCR. This work proves the validity of adenoma-specific markers across multi-populations, which would contribute to the early diagnosis and treatment of CRC.

## Abstract (web summary)

The gut microbiome plays an important role in colorectal carcinogenesis and predictive microbiome signatures have been proposed for colorectal cancer (CRC) diagnosis. Here the authors perform a meta-analysis of 16S rRNA-based profiles to identify microbial markers able to discriminate patients with adenoma from control and CRC, building a model that can be applied for the early detection of CRC.

## Introduction

Colorectal cancer (CRC) is one of the most common cancers with an overall high mortality rate. According to the report of the International Agency for Research on Cancer (IARC), there were over 1,800,000 new CRC cases and over 860,000 deaths in 2018^1^. And CRC accounted for approximately 10% of all new cancer cases globally^2^. It is estimated that the national expenditures in the United States on cancer care, specifically colorectal cancer, were about 16.63 billion dollars in 2018^3^, and the CRC burden is continuously growing over years. Colorectal adenomas are recognized as precursors for the majority of CRC^2^. The early detection of CRC at precancerous-stage adenoma has brought the 5-year relative survival rate to around 90%, significantly facilitating early decision making, alleviating the incidence of CRC, and reducing economic burden^2,4^.

Gut microbiome is a stool-based non-invasive biomarker for metabolic diseases and cancers^5,6^. Many studies have reported that the gut microbiome is an important etiological element in the initiation and progression of CRC^4,7^ and have identified some fecal microbial markers of CRC^8–10^. However, it is not clear whether these biomarkers could precisely detect adenomas, early-stage CRC. Furthermore, current knowledge of the associations between the microbiome and colorectal adenoma is limited. Only a few studies have investigated the microbial alterations in colorectal adenoma^4,7,11–13^. Besides, substantial variations on microbial makers exist among these studies, which could be due to various biological factors influencing gut microbiome composition and inconsistent processing of microbial sequencing data.

Meta-analysis offers a set of tools that are powerful, informative, and unbiased to reduce the noise of biological and technical confounders so that consistent and robust alterations across multiple studies could be identified. Recently, several meta-analyses on multi-studies have identified universal microbial markers across multiple diseases, such as CRC^11,13–15^, obesity^16^, inflammatory bowel disease (IBD)^17^, via 16S rRNA sequencing or whole metagenome shotgun sequencing (WMS) technique. However, universal microbial markers specific for colorectal adenoma were less frequently reported or showed relatively lower accuracies for diagnosis^11,13^. Thomas et al^11^ identified a few microbial markers of colorectal adenomas from a WMS-based meta-analysis and their classifiers showed low accuracy in distinguishing adenomas from healthy controls (area under the ROC curve (AUC) = 0.54) or CRC (AUC = 0.69)^11^, probably due to the limited coverage of taxonomy and high dependence on reference genomes in WMS taxonomic profiling^18^. A recent meta-analysis study based on 16S rRNA mainly investigated colonic cancerous tissues and identified some tissue-based microbial markers for colorectal adenoma^13^. Tissue-based microbial markers were invasive and less accessible than stool-based microbial markers. Additionally, the commonly used non-invasive stool-based screening test, fecal immunochemical test (FIT), has drawbacks such as poor sensitivity to early and advanced adenoma (7.6% and 38%, respectively)^19^. Therefore, it is urgent to explore and identify stool-based microbial markers that could more precisely and efficiently diagnose colorectal adenoma.

In this work, we perform an integrated analysis on a total of 1056 samples with published 16S rRNA data from multiple studies considering that 16S rRNA-based profiles are better representations of the “real community”^20^. Based on the discovery dataset comprising 775 samples, we construct the Random Forest (RF) model achieving a high accuracy (AUC = 0.80) with 11 important features to distinguish colorectal adenoma from non-tumor control. Similarly, the AUC of the RF model for distinguishing colorectal adenoma from CRC with 26 important features is 0.89. Through study-to-study transfer validation and leave-one-dataset-out (LODO) validation across multiple data sets, the important features can overcome technical and geographical discrepancies with an average AUC of 0.76 in the adenoma-control model and 0.89 in the adenoma-cancer model. These important features are validated with two additional independent cohorts comprising 281 samples and are specific to adenoma against other microbiome-linked diseases. Furthermore, pooled functional analysis based on the Phylogenetic Investigation of Communities by Reconstruction of Unobserved States (PICRUSt2) reveals that altered microbiome is characterized by increased ADP-l-glycero-beta-d-manno-heptose (ADP-heptose) biosynthesis in adenoma and elevated menaquinol-10 (MK-10) biosynthesis (*P* < 0.05) in CRC. These findings are validated with a newly collected cohort of 43 samples using quantitative real-time PCR (qRT-PCR). The integrated analyses of heterogeneous studies prove the validity of adenoma-specific markers across multi-populations, which would contribute to the early diagnosis and treatment of CRC.

## Results

### Characteristics of the data sets in meta-analysis

In this study, we investigated 16S rRNA sequencing data from four studies to evaluate the gut microbiome changes as CRC progresses (from control to adenoma to cancer) and to identify the biomarkers specific to adenoma. In total, we collected 306 samples from colorectal adenoma patients, 217 from CRC subjects, and 252 samples from healthy controls. The demographic information was listed in Table 1. All samples were sequenced at sufficient depth except one sample in US1 (SRR5184891), which was excluded for further analysis. The average count of sequencing reads in each sample is 85,637. Consistent processing was performed for all raw sequencing data on the Quantitative Insights Into Microbial Ecology 2 (QIIME2) platform.

**Table 1:** Characteristics of the large-scale adenoma data sets included in this study.

Study	Group (*N*^a^)	Age (average ± s.d^b^)	BMI (average ± s.d^b^)	Sex F(%)/M(%)^c^	No. of reads (average ± s.d^b^)	Country
CA^12^	Control (30)	55.27 ± 9.22	26.73 ± 5.19	63.30/36.70	109,885 ± 55,194	USA Canada
	Adenoma (30)	61.30 ± 11.15	27.40 ± 4.45	60.00/40.00		
	Cancer (30)	59.40 ± 10.99	30.59 ± 7.18	70.00/30.00		
FR^21^	Control (50)	62.32 ± 8.98	24.66 ± 4.69	52.00/48.00	215,465 ± 119,217	France
	Adenoma (38)	62.29 ± 8.51	27.40 ± 4.45	28.90/71.10		
	Cancer (41)	65.51 ± 10.51	30.59 ± 7.18	41.50/58.50		
US1^40^	Adenoma (40)	62.33 ± 9.12	26.22 ± 4.22	37.50/62.50	48,337 ± 25,069	USA
	Cancer (26)	61.65 ± 12.89	28.63 ± 7.19	42.30/57.70		
US2^27^	Control (172)	54.29 ± 9.93	26.69 ± 5.33	64.50/35.50	52,028 ± 36,596	USA
	Adenoma (198)	63.35 ± 11.47	26.27 ± 4.73	40.40/59.60		
	Cancer (120)	63.78 ± 12.89	28.89 ± 7.25	43.30/56.70		
	Control (252)	56.00 ± 10.14	26.48 ± 5.25	61.90/38.10		
Total	Adenoma (306)	62.89 ± 10.80	26.21 ± 4.80	38.89/61.11	85,637 ± 85,460	
	Cancer (217)	63.25 ± 12.28	28.30 ± 7.23	41.01/58.99		

All data sets were sequencing data of the V4 region of the 16S rRNA gene.

^a^Number of samples.

^b^Standard deviation.

^c^The ratio of the percentage of female and male.

### Identification of the potential confounder in meta-analysis

**Fig. 1:**
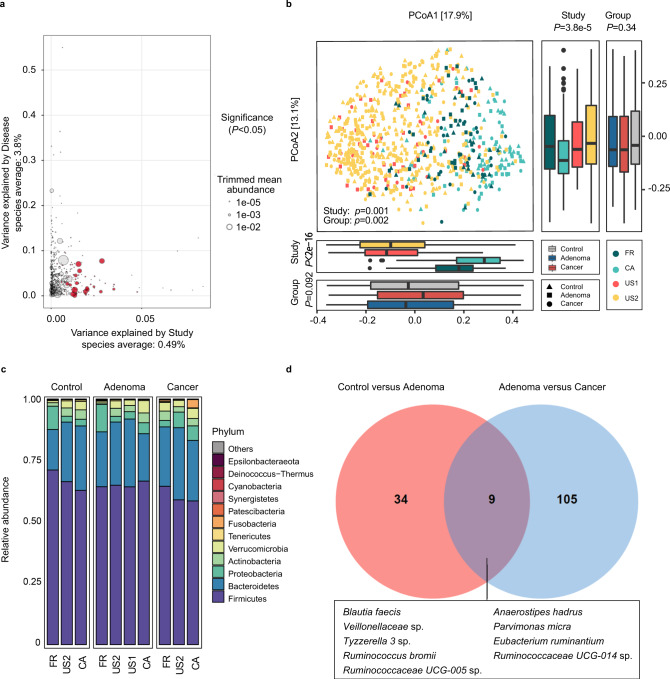
Alterations of gut microbial composition in different disease status. **a** Variance explained by disease status (adenoma versus cancer) is plotted against variance explained by study effects for individual ASVs. The significantly differential ASVs are colored in red and the dot size is proportional to the abundance of each ASV. *P* values were from a two-sided blocked Wilcoxon rank-sum test. Source data and exact *P* values are provided as a Source Data file. **b** Principal coordinate analysis of samples (control, *n* = 252; adenoma, *n* = 306; cancer, *n* = 217) from all four studies based on Bray–Curtis distance, which shows the fecal microbiota composition was different among studies (*P* = 0.001) and groups (*P* = 0.002). *P* values of beta diversity based on Bray–Curtis distance were calculated with PERMANOVA. The study is color-coded and the group (control, adenoma, and cancer) is indicated by different shapes. The upper-right and the bottom-left boxplots illustrate that samples projected onto the first two principal coordinates broken down by study and disease status, respectively. *P* values of the first and second principal components were calculated with a two-sided Kruskal–Wallis test for study and group. All boxplots represent the 25th–75th percentile of the distribution; the median is shown in a thick line at the middle of the box; the whiskers extend up to values within 1.5 times of IQR, and outliers are represented as dots. Source data are provided as a Source Data file. **c** Relative proportions of bacterial phyla in healthy controls, adenomas, and CRC across four different studies. **d** Venn diagram shows the overlap of differential ASVs assigned at species level between adenomas and healthy controls or CRC.

Since differences existed among these studies in both technical and biological aspects, we first investigated the potential confounders. The variances explained by disease status for each amplicon sequence variants (ASVs) were calculated to quantify the effects of potential confounders (see “Confounder analysis” section, Fig. 1a and Supplementary Fig. 1, 2). The variance of ASVs explained by “study” was greater than that by disease status and by other potential confounders. Additionally, beta diversity varied among different studies (*P* = 0.001, Fig. 1b). These results revealed that the factor “study” had a predominant impact on microbial composition at both the single taxon level and community level. Therefore, we treated “study” as a blocking factor in the subsequent analysis and used a two-sided blocked Wilcoxon rank-sum test to adjust the batch effect and identify differential ASVs that were less affected by “study”.

### Alterations of gut microbial composition in colorectal adenoma

Gut microbiota highly varied among different disease statuses (*P* = 0.002, Fig. 1b). Moreover, the Shannon index showed no significant differences between groups (Supplementary Fig. 3a), while the Simpson’s Index of Diversity was significantly higher in the adenoma groups (*P* = 0.043) and in the control groups (*P* = 0.020, Supplementary Fig. 3b) than that in the cancer groups when blocking the “study” confounder.

At the phylum level, the gut microbiota was dominated by members of Firmicutes and Bacteroidetes, followed by Proteobacteria, Actinobacteria, Verrucomicrobia, Tenericutes, and Fusobacteria in healthy controls, adenomas, and CRC (Fig. 1c). These dominant phyla were similar to those reported in the previous studies^21^. Furthermore, the phylum Fusobacteria, the most CRC-associated bacteria as reported^22^, had significantly decreased abundance (*P* < 0.05) in adenoma compared to that in cancer, while showed no significant difference between adenoma patients and controls (Fig. 1c and Supplementary Data 1).

At the ASV level, 43 ASVs were identified with distinguishable differential abundances in the comparison of gut communities between controls and patients with adenoma. Specifically, there were six ASVs depleted in adenoma, which were assigned as *Bifidobacterium longum*, *Anaerostipes hadrus*, *Lactococcus taiwanensis*, *Aminipila butyrica*, etc. Besides, the abundances of 37 ASVs were increased in adenoma compared with control, and they were assigned as *Eubacterium coprostanoligenes*, *Methanobrevibacter millerae*, *Christensenellaceae R-7* group sp., etc (Supplementary Data 2). Moreover, we also identified 114 differentially abundant ASVs between adenoma and cancer. Among these, 56 ASVs were in lower abundance in adenoma compared with cancer, which were assigned as *Lachnoclostridium* sp., *[Ruminococcus] gnavus* group sp., *[Clostridium] scindens*, *Escherichia-Shigella* sp., etc. The ASVs in higher abundance in adenoma than cancer were assigned as *Blautia obeum*, *Butyricicoccus faecihominis*, *Erysipelotrichaceae UCG-003* sp., *Dorea longicatena*, etc (Supplementary Data 3).

Additionally, pathogenic bacteria with increased abundance were detected in adenoma or cancer compared with control. For instance, ASVs assigned as *Parvimonas micra* was enriched in adenoma compared with control (Supplementary Data 2) while ASVs assigned as *Fusobacterium nucleatum*, *Porphyromonas* sp. *HMSC077F02*, *Porphyromonas asaccharolytica, Peptostreptococcus stomatis*, *P. micra*, and *Escherichia-Shigella* sp. were enriched in cancer compared with adenoma (Supplementary Data 3). Notably, between control versus adenoma and adenoma versus cancer, there were only nine common differential ASVs, which were assigned as *Blautia faecis*, *A. hadrus*, *P. micra*, *Tyzzerella 3* sp., *Eubacterium ruminantium,* etc (Fig. 1d). The two sets of differential ASVs with a Jaccard distance of 0.939 indicate that the microbiota has a remarkable difference between adenoma and control or cancer.

### Microbial classification models for colorectal adenoma

Next, we constructed stratified 10-fold cross-validation RF models, by pooling all samples to distinguish adenoma from control and cancer. Besides using differential ASVs as key metrics, alpha diversity indices including Shannon Index, Simpson Index, and Observed ASVs, and three patient metadata, age, sex, and body mass index (BMI) were also included in model building. To obtain the best performing models and important features, an iterative feature elimination (IFE) step was further applied.

**Fig. 2:**
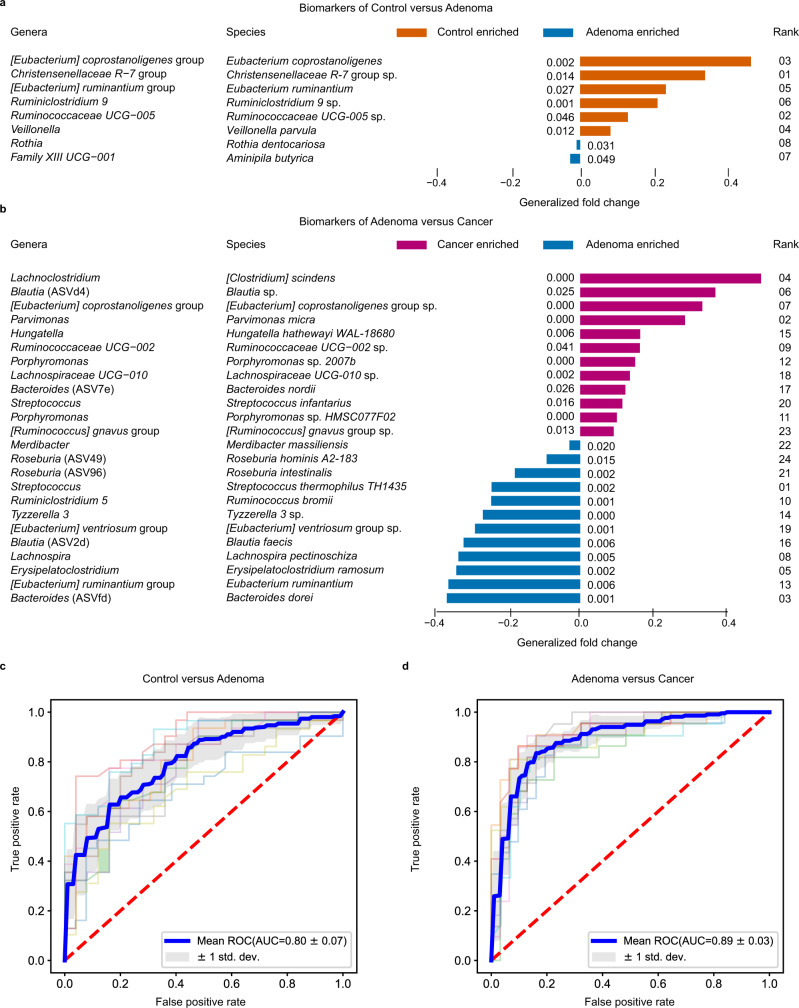
Performance of discriminating adenoma from control or cancer using important features. **a**, **b**, The biomarkers were identified to construct RF models for discriminating adenoma from control (**a**) and CRC (**b**). Each biomarker represented a single ASV, and the genera and species columns displayed the taxonomy information for the ASVs at the genus and species level. The rank in **a** and **b** indicates the order of feature importance in the RF model; *P* values were computed using a two-sided blocked Wilcoxon rank-sum test and the exact *P* values were presented beside the barplots. Generalized fold change (see Methods meta-analysis of differentially abundant ASVs) was indicated by color gradients. Source data are provided as a Source Data file. **c**, **d** The AUC of the optimized models constructed with biomarkers and patient metadata of control versus adenoma (**c**) and adenoma versus cancer (**d**). Mean AUC and standard deviation of stratified 10-fold cross-validation were shown in **c** and **d**.

A robust RF model was eventually constructed with a core set of important features, including eight differential ASVs (as biomarkers) together with age, sex, and BMI, which achieved an AUC of 0.80 for distinguishing control subjects from adenoma patients (accuracy: 0.73, sensitivity: 0.82, specificity: 0.62, precision: 0.73 and F1 score: 0.77, Fig. 2a, c, Supplementary Data 4, and Supplementary Table 1). Among these, the ASV assigned as *Christensenellaceae R-7* group sp. was the highest-ranking biomarker (Fig. 2a). The biomarkers also included ASVs assigned as *E. coprostanoligenes*, *Ruminiclostridium 9* sp., *Christensenellaceae R-7* group sp., *Ruminococcaceae UCG-005* sp., and *Veillonella parvula* of increased abundance as well as *Rothia dentocariosa* and *A. butyrica* of decreased abundance in adenoma (Fig. 2a).

Similarly, the RF model in distinguishing adenoma from cancer achieved an AUC of 0.89 (accuracy: 0.80, sensitivity: 0.66, specificity: 0.90, precision: 0.83 and F1 score: 0.72, Fig. 2b, d and Supplementary Table 1). The RF model was built with 24 ASVs together with age and BMI (Fig. 2b and Supplementary Data 5). Among these, the ASV belonging to *Streptococcus thermophilus TH1435* was the top-ranking biomarker (Fig. 2b), followed by ASVs assigned as *P. micra*, *Bacteroides dorei*, *C. scindens*, *Erysipelatoclostridium ramosum*, *Blautia* sp., *[Eubacterium] coprostanoligenes* group sp., and *Lachnospira pectinoschiza* (Fig. 2b). The *C. scindens* was significantly (*P* < 0.001) enriched in cancer compared with adenoma. Additionally, the abundance of ASVs assigned as *C. scindens*, *Blautia* sp., *[Eubacterium] coprostanoligenes* group sp. and *P. micra* increased in CRC while *S. thermophilus TH1435*, *E. ruminantium*, *E. ramosum* and *L. pectinoschiza* increased in adenoma (Fig. 2b). In these two models, age was ranked as the top and third predictor in the testing phase, respectively. In the two sets of biomarkers, there was only one common ASV classified as *E. ruminantium*.

Moreover, we also identified that a core set of 34 ASVs, together with age, sex, and BMI, collectively had the highest capability to distinguish control from cancer (AUC = 0.93, Supplementary Fig. 4). The ASVs ranked as the top important markers assigned as *F. nucleatum* and *P. asaccharolytica*, which were also ranked as top markers in two recent meta-analysis of CRC based on WMS data (Supplementary Data 6). Moreover, we found that there were six common biomarkers between CRC-vs-control biomarker set and CRC-vs-adenoma biomarker set, while there was no common ASV in the two sets of biomarkers between control-vs-adenoma and control-vs-CRC (Supplementary Fig. 5). These results highlighted that microbial markers aimed to detect CRC are specific and exclusive, not as applicable for diagnosing adenoma.

### Co-occurrence and clustering analysis of microbiota

We next constructed the co-occurrence network of differential ASVs, using the SparCC algorithm^23^. In the co-occurrence network of differential ASVs between adenoma and control, we found widespread negative correlations among these ASVs, indicating a status of many competitions among community members in an unstable community (Supplementary Fig. 6a and Supplementary Data 7). Notably, most of the negative correlations were associated with the ASV assigned as *A. hadrus* (the 2nd ASV), which may protect against colon cancer in humans by producing butyric acid^24^. The first and second ranking biomarkers between adenoma and control, assigned as *Christensenellaceae R-7* group sp. and *Ruminococcaceae UCG-005* sp., were highly correlated to other ASVs, indicating important roles in the microbial community. Moreover, a module containing 8 nodes and 15 interactions was identified by MCODE^25^ with the highest score (Supplementary Fig. 6b). In this module, the biomarker assigned as *Ruminococcaceae UCG-005* sp. acted as the hub node, and associated with a wide range of ASVs assigned as *R. dentocariosa*, *A. hadrus* (the 2nd ASV), *Ruminococcaceae UCG-002* sp. (the 15th ASV), and *B*. *longum* (the 1st ASV).

Additionally, we constructed the co-occurrence network of differential ASVs between adenoma and CRC (Supplementary Fig. 6c and Supplementary Data 8). Positive correlations among the adenoma- and CRC-enriched ASVs were observed in general while negative correlations were also observed. Two modules were identified by the MCODE from this network (Supplementary Fig. 6d). One module comprised 14 nodes and 72 edges with a score of 11.08. In this module, the top-ranking biomarker, *S. thermophilus TH1435* was correlated with multiple nodes, such as *[Ruminococcus] gnavus* group sp., *[Eubacterium] nodatum* group sp. (the 62nd ASV), and *Faecalibacterium prausnitzii A2-165* (the 24th ASV). The other module contained five nodes and 10 edges, in which the biomarker assigned as *C. scindens* was capable of converting primary bile acids to toxic secondary bile acids inducing cancer^26^. In summary, our results suggested that most of the identified biomarkers have a broad and large impact on the members of the microbial networks.

To gain further insight, we analyzed and compared the pattern of biomarkers in adenoma and control groups, which were further assembled into four clusters with distinct taxonomic compositions (Supplementary Fig. 7a). These clusters are not tightly associated with patient characteristics such as age, sex, and BMI (Supplementary Fig. 8a). Moreover, we also explored the CRC patient gut microbiota for co-occurrences among a panel of 24 biomarkers and yielded three clusters (Supplementary Fig. 7b). Cluster 1 had the fewest ASVs that were assigned as species from Lachnospiraceae family, and cluster 2 was heterogeneous in taxonomy with a relatively high prevalence in CRC individuals. Notably, cluster 3 demonstrated strong taxonomic consistency, primarily belonging to Clostridiales. We then investigated associations of these clusters with various tumor characteristics. These biomarker clusters were not biased by patients’ age, BMI or cancer stage, but cluster 1 was significantly enriched in female CRC patients. (Supplementary Fig. 8b). Considering the impact of different studies, all of these tests were adjusted by blocking “study” (see “Co-occurrence and clustering analysis” section).

### Validation of the colorectal adenoma classifiers

To test whether the identified important features are universal and robust across multiple studies, we performed study-to-study transfer validation and LODO validation on the entire samples.

**Fig. 3:**
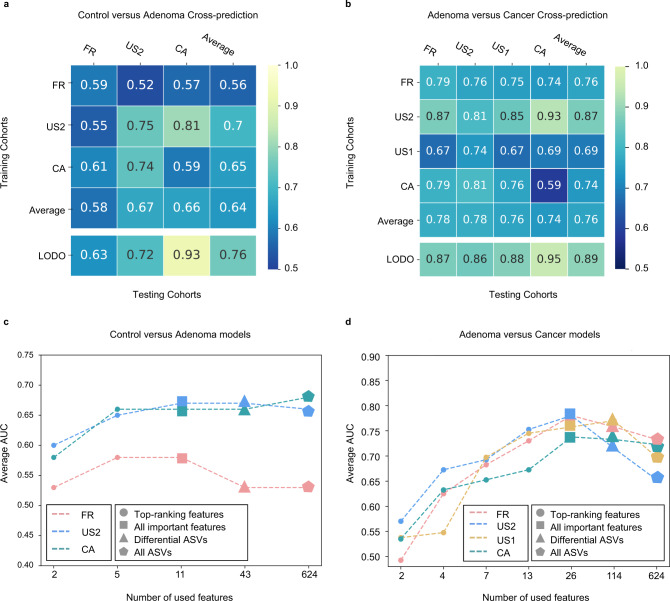
Prediction performance of important features across studies and identification of minimal features for detecting adenoma. **a**, **b**, Cross-prediction matrix detailing prediction values for differentiating adenoma from control using bagging K-Nearest Neighbors classifiers (**a**) and CRC using RF models (**b**) as AUC obtained using important features. Values on the diagonal refer to the results of cross-validation within each study. Off-diagonal values refer to the AUC values obtained from cross-cohort validation, which training the classifier on the study of the corresponding row and applying it to the study of the corresponding column. The LODO values refer to the performances obtained by training the classifier using all but the study of the corresponding column and applying it to the study of the corresponding column (see “Model evaluation” section). The study-to-study and LODO validation values for differentiating adenoma from control using RF models can be found at Supplementary Fig. 9. **c**, **d** Average AUC of study-to-study transfer validation classifiers for control versus adenoma (**c**) and adenoma versus cancer (**d**) with different sets of features. Input features were indicated as different shapes, top-ranking features, all important features signed, differential ASVs and all ASVs were represented by circles, squares, triangles, and pentagons, respectively. The *x* axis in **c** and **d** indicate different numbers of features. Colors represent different studies. Source data are provided as a Source Data file.

In the control versus adenoma models, the AUC values of study-to-study transfer validation ranged from 0.52 to 0.81, with an average of 0.64 (Fig. 3a). Notably, the US2 study served as a better training set than other studies achieving relatively higher testing AUCs (average AUC = 0.70). This may be explained by the larger size of the dataset. Moreover, to compare the diagnostic performance of the important features with the FIT, the most widely used non-invasive stool test, we collected the publicly available FIT samples (including 172 control individuals and 198 adenoma patients) from a published study^27^. The performance of the RF model constructed with FIT being the only feature for distinguishing adenoma from control is 0.60 (AUC). The model constructed with important features tested on the cohorts in this study was proved to be superior to that of the FIT, with an AUC of 0.78. Moreover, the combination of FIT with the important features further improved the diagnostic accuracy for adenoma (about 3%) and achieved the best performance of 0.81 (AUC) (Supplementary Fig. 10). Altogether, our results demonstrate that the microbial-derived biomarker panel is superior to FIT for detecting colorectal adenoma and their combination can improve the accuracy of non-invasive diagnosis of adenoma. Additionally, the AUC values of LODO analysis ranged from 0.63 to 0.93 (average AUC = 0.76), which was better than those achieved in study-to-study transfer validation owing to using a larger amount of training data (Fig. 3a). Furthermore, with the increase of training samples, the AUC values of LODO analysis increased in parallel (Supplementary Fig. 11), predicting a trend of improved diagnostic accuracy as more public adenoma data sets become available.

Similar results were observed in the adenoma versus cancer models (Fig. 3b). The AUC values of study-to-study transfer validation ranged from 0.59 to 0.93 (average AUC = 0.76). Moreover, the AUC values were also elevated in the LODO analysis, ranging from 0.86 to 0.95 with an average of 0.89 (Fig. 3b). Additionally, control versus cancer models showed robustness through study-to-study transfer validation (average AUC = 0.83) and LODO validation (average AUC = 0.90) (Supplementary Fig. 12). We noticed that the classifiers performed better in adenoma versus cancer and control versus cancer than that in control versus adenoma, likely because the adenoma-associated stool microbiome closely resembles that of the healthy status^7,11,21^.

Furthermore, we tested the diagnostic capability of several sets of features including all ASVs, differential ASVs and all important features (Supplementary Fig. 13). In both study-to-study transfer validation (Fig. 3c, d) and LODO validation (Supplementary Fig. 14a, b), the set of all important features performed better than the other two sets of ASVs, except for the CA study. This may be due to the small sample size and geographic heterogeneity in the CA study. When the number of top-ranking features decreased, the accuracy of classifiers decreased conformably (Fig. 3c, d). Therefore, these results supported the use of all important features as the main feature set for adenoma diagnosis.

### Validation of colorectal adenoma markers in independent cohorts

To further validate our meta-analysis results, two additional independent cohorts from America (validation cohort1) and China (validation cohort2) were incorporated into this study. The validation cohort1 is comprised of 70 controls and 102 adenoma patients, while there are 57 adenoma patients and 52 CRC patients in the validation cohort2 (Supplementary Table 2). The reconstructed RF models in the two independent cohorts achieved AUCs of 0.78 (accuracy: 0.70, sensitivity: 0.76, specificity: 0.59, precision: 0.71 and F1 score: 0.77) and 0.84 (accuracy: 0.79, sensitivity: 0.79, specificity: 0.80, precision: 0.78 and F1 score: 0.72) for distinguishing adenoma from controls or cancer, respectively (Supplementary Fig. 15a, b). Notably, only microbial biomarkers and sex information were used in the validation cohort2 due to the unavailability of age and BMI information, which achieved a relatively higher AUC. Additionally, the features’ ranks were consistent with that in the discovery RF models, for instance, ASVs assigned as *Ruminococcaceae UCG-005* sp. and *Christensenellaceae R-7* group sp. were confirmed as the top-ranking biomarkers between controls and adenoma patients in validation cohort1 (Supplementary Data 9). Furthermore, ASVs assigned as *P. micra*, and *B. dorei* were also confirmed as the top-ranking biomarkers for distinguishing between adenoma and CRC patients in validation cohort2 (Supplementary Data 10).

### The specificity of colorectal adenoma predictive models

Since improving the specificity of markers could reduce false positives in clinical diagnosis^17^, it is necessary to further evaluate the specificity of our identified adenoma markers, such as in the context of other microbiome-linked diseases^11^. In this analysis, five non-CRC diseases including Crohn’s disease (CD), ulcerative colitis (UC), irritable bowel syndrome (IBS), non-alcoholic fatty liver disease (NAFLD), and type 2 diabetes (T2D) were considered (Supplementary Table 2). The AUC values of non-CRC disease models were significantly lower than that of an independent adenoma model (Supplementary Fig. 16), which indicated that our markers have high specificity for adenoma.

### Microbial functional changes in colorectal adenoma

**Fig. 4:**
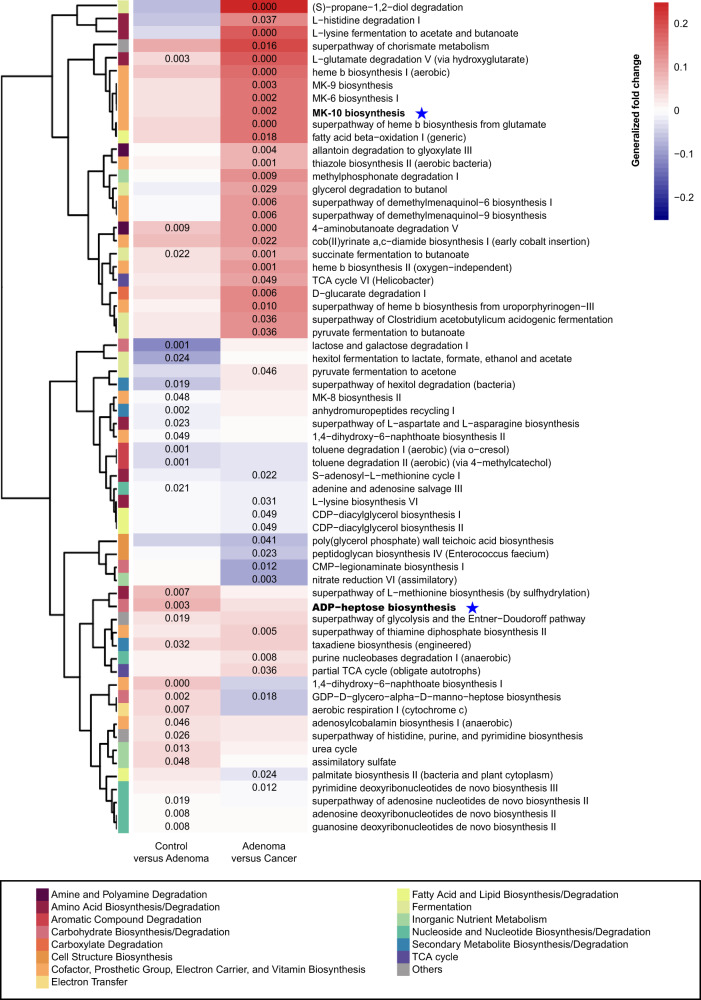
Functional alterations in control, adenoma, and cancer. The relative abundances of functional pathways were compared between adenoma and control or cancer. Differentially abundant pathways were plotted; *P* values were computed using a two-sided blocked Wilcoxon rank-sum test and the exact *P* values < 0.05 were presented in the heatmap. Generalized fold change (see “Meta-analysis of differentially abundant ASVs” section) was indicated by color gradients. The generalized fold change > 0: enriched in the latter; generalized fold change < 0: enriched in the former. Source data are provided as a Source Data file.

We examined the microbiome-based functional alterations on multiple different disease status. There were 27 differential pathways between control and adenoma (Supplementary Data 11) and 41 differential pathways between adenoma and cancer (Supplementary Data 12) consistently detected across studies. A total of 64 differential pathways (4 pathways were overlapped) were clustered based on their generalized fold change scores (Fig. 4). In detail, in comparison between adenoma and control, pathways of carbohydrate biosynthesis (e.g., ADP-heptose biosynthesis), inorganic nutrient metabolism, and nucleoside and nucleotide biosynthesis were enriched in adenoma, whereas, pathways of aromatic compound degradation, and secondary metabolite biosynthesis were decreased in adenoma samples. In comparison between adenoma and CRC, pathways of cofactor, prosthetic group, electron carrier, vitamin biosynthesis (e.g., MK-10 biosynthesis), and amino acid degradation and fermentation were enriched in cancer. On the other hand, cell structure biosynthesis and fatty acid and lipid biosynthesis/degradation pathways were decreased in adenoma.

**Fig. 5:**
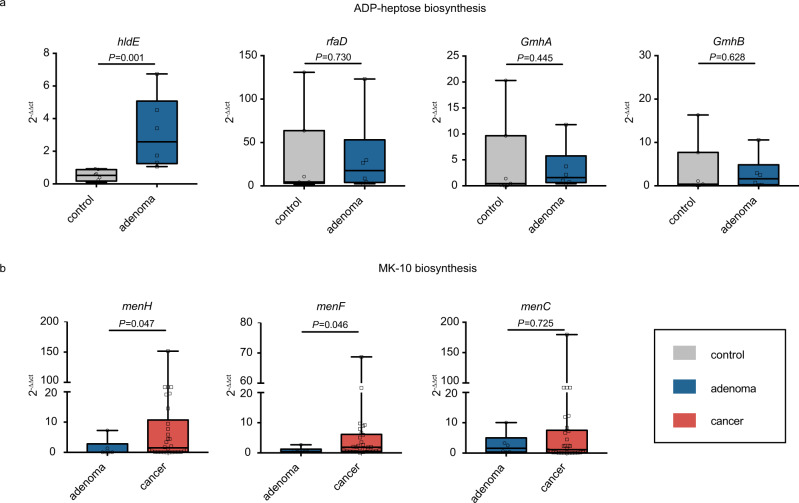
Relative abundance of candidate genes. Plotted values are qRT-PCR quantifications of bacterial genes in the ADP-heptose and MK-10 biosynthesis. The abundances of **a** *hldE*, *rfaD*, *GmhA*, and *GmhB* were compared between control (*n* = 7) and adenoma (*n* = 6) groups, while the abundances of **b** *menH*, *menF,* and *menC* were compared between adenoma (*n* = 6) and cancer (*n* = 30) groups. All boxes extend from 25th to 75th percentiles and whiskers show the minimum and maximum values. Lines at the middle of each box show the median. *P* values were computed using a two-sided Wilcoxon rank-sum test. Source data are provided as a Source Data file.

Notably, the abundance of biosynthesis of ADP-heptose, a key metabolic intermediate in the biosynthesis of lipopolysaccharide (LPS) was significantly enriched in adenoma compared with control. It was associated with the activation of the nuclear factor-κB (NF-κB) and a strong pro-inflammatory response^28^, which led to colorectal adenoma. The ASV assigned as *V. parvula*, one of the biomarkers differentiating healthy controls from adenoma samples (Fig. 2a), was a major contributor to the ADP-heptose biosynthesis (ranked 9 out of 624 in adenoma patients and ranked 16 in controls, Supplementary Data 13). There are four rate-limiting enzymes encoded by *hldE*, *rfaD*, *gmhA*, and *gmhB* in the biosynthesis of ADP-heptose. These four genes were consistently enriched in adenoma compared with control (Supplementary Table 3). Further, we validated the abundance of these key genes based on qRT-PCR using newly collected samples. Consistent with the PICRUSt2 results, *hldE* and *rfaD* genes were enriched in adenoma compared with control (Fig. 5a), especially that the abundance of *hldE* gene was significantly increased in adenoma.

Moreover, it was worth noting that menaquinone (vitamin K2) biosynthesis was significantly enriched in cancer compared with adenoma, especially the MK-10 biosynthesis. MK-10 was mainly produced by the ASV assigned as *B. dorei*, one of the biomarkers between adenoma and cancer (Fig. 2b), and was the 3rd and 4th contributor to MK-10 biosynthesis in adenoma and cancer among all ASVs (Supplementary Data 14). Collectively, the elevated production of vitamin K2 by microbiota may serve as a response to compensate for the induction of feedback inhibition in colorectal cancer cells^29^. Furthermore, we found a significantly increased abundance of *menH*, *menF,* and *menC* in CRC samples compared with that of adenoma in pooled data sets by a two-sided blocked Wilcoxon rank-sum test (Supplementary Table 4). These results were also confirmed by qRT-PCR with our newly-collected samples (Fig. 5b), showing that *menH* and *menF* genes were significantly increased in the CRC samples than those in the adenoma samples.

## Discussion

This study comprehensively assessed the alterations of CRC-associated gut microbiome and the capability of microbial markers for early detection of CRC at precancerous-stage adenoma. The best performing model achieved a high accuracy (AUC = 0.80) with 11 important features to distinguish colorectal adenoma from non-tumor control (Fig. 2c). Similarly, the AUC of the best model for detecting colorectal adenoma from CRC with 26 important features was 0.89 (Fig. 2d). Through study-to-study transfer validation and LODO validation across multiple data sets, the important features could overcome technical and geographical discrepancies with an average AUC of 0.76 in the adenoma-control model (Fig. 3a) and 0.89 in the adenoma-cancer model (Fig. 3b). These important features were validated with two additional independent cohorts (Supplementary Fig. 15a, b) and were specific to adenoma against other microbiome-linked diseases (Supplementary Fig. 16).

It has long been reported that fecal bacteria could serve as biomarkers for non-invasive diagnosis of CRC, such as *F. nucleatum*, *Escherichia coli*, and *Bacteroides fragilis*^8,30–32^. However, large variations existed among studies for these microbial markers^17^, indicating the necessity of multi-cohort integration analysis. Two pioneering studies^11,14^ have performed cross-cohort analyses focusing on distinguishing CRC patients from controls based on WMS data. In contrast, our study aimed at identifying adenoma-specific microbial markers, because early screening of CRC is of the paramount value for the patients. In Thomas’s work, adenoma-related classifiers showed lower accuracies in distinguishing adenomas from healthy controls (AUC = 0.54) or CRC (AUC = 0.69)^11^. One explanation is that the adenoma-associated stool microbiome closely resembles that of the health status^7,11,21^. Besides, it is probably also influenced by the limited coverage of taxonomy and the high dependence on reference genomes in WMS taxonomic profiling^20,33^. WMS data is well-recognized to possess the advantage of species- and even strain-level resolution. However, the current strategies for characterizing microbial community compositions with WMS are “closed annotation” that strongly rely on the known reference genome database^18,34,35^, which is likely missing some species without known genomes or marker genes. It will thus result in biases in relative abundance estimation. Consequently, in this study, we included fecal 16S rRNA sequencing studies considering that 16S rRNA gene-based profiles are better representations of the “real community”^20^. Moreover, considering inconsistent abundance changes among ASVs assigned as the same species, we constructed classifiers at the ASV level to capture the most informative ASVs that could effectively distinguish patients from controls. The control-CRC model built in this study with 16S rRNA profiling achieved an AUC of 0.93, whose accuracy was significantly higher than that based on WMS (AUC = 0.84)^11,14^. Similarly and more importantly, we constructed models using sets of microbial markers that distinguish colorectal adenoma from controls (AUC = 0.80) and CRC (AUC = 0.89) with high accuracy. These markers were validated for effectiveness via study-to-study transfer validation and LODO validation as well as with independent cohorts. Furthermore, we confirmed that the identified panel of markers was colorectal adenoma-specific rather than other microbiome-associated diseases, such as IBD and NAFLD (Supplementary Fig. 16). Overall, all these validations strongly support the robustness of the classifiers and provided evidence that stool-based microbial markers could serve as an effective non-invasive clinical indicator for colorectal adenoma.

Microbial communities varied in both colorectal adenoma and cancer during the progression of CRC. A large-cohort CRC study revealed distinct stage-specific shifts of microbiome and metabolome and found elevated *Atopobium parvulum* in adenoma compared to controls^15^. Notably, we also found that both differential ASVs and markers for distinguishing adenoma and cancers from healthy controls varied greatly. The ASV assigned as *E. ruminantium* was the only common adenoma-associated marker while *Porphyromonas* sp. *HMSC077F02*, *L. pectinoschiza*, *Hungatella hathewayi WAL-18680*, etc were common cancer-associated biomarkers. *F. nucleatum*, one of the universal biomarkers in our cancer-control model and the two recent CRC meta-analysis^11,14^, was neither a differential bacterium nor a biomarker between controls and adenomas. In addition, prior work indicated that the diagnostic capability of *Fusobacterium* sp. for colorectal adenoma was inferior to that of strain “*m3*” of the *Lachnoclostridium* sp.^4^. These results indicated that the CRC-associated biomarkers were not effective for the detection of colorectal adenoma and highlighted the importance of adenoma-specific signatures. Additionally, the adenoma-specific markers may contribute to the early screening and consequently reduce the risk of CRC. What’s more, the combination of the important adenoma-specific markers and FIT improved the classifier’s accuracy (AUC = 0.81) compared to microbial makers (AUC = 0.78) or FIT (AUC = 0.60) alone (Supplementary Fig. 10), indicating that the non-invasive FIT test could be used as complementary tool to gut microbiota analysis for early screening of adenoma. Recently, a 16S rRNA analysis investigated microbiome dysbiosis in adjacent tissues of colonic cancerous tissue and the identified signatures could discriminate colorectal adenomas from healthy controls effectively^13^, though tissue-based markers are invasive and less accessible than stool-based markers.

The functional analysis sheds light on the convoluted underlying mechanisms and would greatly enhance our understanding and interpretation of CRC carcinogenesis (Supplementary Fig. 17). In particular, we found that the biosynthesis of ADP-heptose and the key gene *hldE* were significantly enriched in adenoma compared with control. ADP-heptose has been identified as a bacteria-linked carcinogen^36^ and the key metabolic intermediate in the biosynthesis of LPS. It is a potent trigger for the activation of NF-κB signaling, which has been shown to promote tumorigenesis^37^ and may be critical in perpetuating inflammation^38^. The increased abundance pattern of ADP-heptose biosynthesis pathway from control to adenoma and to CRC suggests that the elevated activity of this pathway may be one important factor that induced the sustained aggravation of NF-κB signaling during the development of CRC. Notably, the pathway abundance of ADP-heptose biosynthesis was significantly increased in adenoma compared to control, while showed no significant enrichment in CRC compared to adenoma. This may suggest that ADP-heptose played a critical role in adenoma and maintained such a role in CRC progression^39^. Moreover, a series of vitamin K2 biosynthesis genes, such as *menH* and *menF* were also significantly different between adenoma and cancer. Previous studies indicated that vitamin K2 played important roles in the antitumor effect via cell-cycle arrest, cell differentiation, and cell apoptosis^29^. Therefore, the increased production of vitamin K2 may be a compensatory effect of the dysregulated microbiota to survive the tumor microenvironment, which also suggests a potential CRC intervention strategy targeting vitamin K2 biosynthesis bacteria. Though the main pathways differed between the control-adenoma and the adenoma-CRC models, all these differential microbial pathways could offer promising perspectives and evidence for intervention and treatment in CRC carcinogenesis.

Being mainly a bioinformatics paper, we recognize the weakness of the study in validation, that is, no intervention study was designed to prove the thesis. To compensate for this weakness, we strived to strengthen the evidence from other perspectives of the study design and provided different types of validations of the identified microbial biomarkers for adenoma, for the purpose of early detection of CRC. Taken together, through extensive and statistically rigorous validation, we identified microbial-derived markers for distinguishing adenoma from healthy control and CRC across multiple studies. Independent validation confirmed that the microbial-derived markers exhibited high accuracy and specificity in detecting adenoma. These microbial-derived markers may contribute to the non-invasive diagnosis of colorectal adenoma and could be targeted to suppress the CRC carcinogenesis. Furthermore, we proposed that the alteration of microbiome-mediated the ADP-heptose biosynthesis activated inflammation in adenoma while the disordered microbiome played a compensatory effect via elevated vitamin K2 production in CRC carcinogenesis.

## Methods

### Public data collection

We collected data from published studies in PubMed.gov containing 16S rRNA sequencing data on patients with CRC, adenomas, and healthy controls. Only four studies with accessible metadata of samples and performance of high-throughput sequencing targeting the V4 region of the 16S rRNA gene were included in this work. Raw sequencing data of these studies were downloaded using SRA toolkit (V.2.9.1) from Sequence Read Archive (SRA) and European Nucleotide Archive (ENA) using identifiers: PRJNA389927 for Zeckular et al.^12^, PRJEB6070 for Zeller et al.^21^, PRJNA290926 for Baxter et al.^27^ and PRJNA362366 for Sze et al.^40^. Besides, two additional cohorts (Supplementary Table 2) were used as independent cohorts with accession numbers PRJNA534511^41^ and PRJNA280026^42^. Sequencing data of four non-CRC studies were utilized to evaluate the specificity of adenoma features. These four data sets were generated from patients who suffered from diseases other than CRC: PRJNA82111^43^, PRJNA544721^44^, PRJEB28350^45^, and PRJNA541332^46^ (Supplementary Table 2).

### Patient recruitment and sample collection

Stool samples were collected from patients with adenoma, CRC, and healthy controls at Fudan University Shanghai Tumor Center with informed consent. Patient recruitment and sample collection were approved by the Medical Ethics Committee of Fudan University Shanghai Tumor Center. Written informed consent was obtained from each participant. This study protocol is in agreement with the world medical association declaration of Helsinki (2008) and the Belmont Report.

Patients were recruited for initial diagnosis and had never received any treatment before fecal sample collection. Patients with hereditary CRC syndromes, and patients with a previous history of CRC were excluded from the study. Based on pathology and colonoscopy results, recruited subjects were classified into three groups: (1) healthy subjects, namely controls: individuals with colonoscopy negative for tumor, adenoma, or other diseases; (2) patients with adenoma: individuals with colorectal adenoma(s); and (3) patients with CRC: individuals with newly diagnosed CRC. A total of 94 subjects were initially recruited. Based on inclusion criteria in addition to similar sex, age, and BMI, 43 samples were enrolled: 30 patients with CRC, 6 adenomas, and 7 controls. The stool was collected in fecal collection tubes and was stored at −80 °C. DNA was extracted from fecal samples using Stool Genomic DNA Kit (CW20925, CWBIO, China) following the manufacturer’s instructions. The patient characteristics for qRT-PCR were summarized in Supplementary Table 5.

### Data preprocessing

The 16S rRNA sequencing data were analyzed using QIIME2 (V.2018.11), a plugin-based platform for microbiome analysis^47^. DADA2 (V.2018.11) software, wrapped in QIIME2, was used to filter out sequencing reads with quality score *Q* > 25 and denoise reads into ASVs (i.e., 100% exact sequence match), resulting in feature tables and representative sequences. Taxonomy classification was assigned based on the naive Bayes classifier using the classify-sklearn package^48^ against the Silva-132-99 reference sequences. ASVs that could not be precisely annotated to species were reassigned to ones having the most similar sequences in the same genus (or family) using NCBI Blast. Subsequently, representative sequences were aligned using Fast Fourier Transform (MAFFT, V.2018.11) in Multiple Alignment and a phylogenetic tree was generated with the Fast-Tree (V.2018.11) plugin. Then, the feature tables were converted to relative abundance tables. A set of ASVs that were confidently detectable in at least three studies and were present in at least 20% of samples was selected for further analysis. One sample (SRR5184891 in PRJNA362366) sequenced at insufficient depth was excluded from the analysis.

### Confounder analysis

We used ANOVA-like analysis^14^ to quantify the effect of potential confounding factors and disease status. The total variance of a given ASV was compared to the variance explained by disease status (control, adenoma, and cancer) and the variance by confounding factors (age, BMI, diabetes, nonsteroidal anti-inflammatory drug (NSAID), platform, race, sex, and study) akin to a linear model. Variance calculations were performed on ranks to account for non-Gaussian distribution of microbiome abundance data^14^. Potential confounding factors with continuous values were transformed into discrete variables either as quartiles or in the case of BMI as groups of lean (>25), overweight (25–30), and obese (>30) based on conventional cutoffs.

### Meta-analysis of differentially abundant ASVs

The significance of differential abundance was tested on a single ASV using a two-sided blocked Wilcoxon rank-sum test implemented in the R (V.3.5.2) “coin” package (*P* values < 0.05 were deemed as significant in all differential analysis). Confounder with high variance explanation was defined as a block to adjust the batch effects in the differential analysis. Significance was tested against a conditional null distribution derived from permutations of the observed data. Permutations were performed within “study” to control variations in block size and composition^14^. For further analysis, we evaluated a generalization of the (logarithmic) fold change for each ASV. This quantity is widely applied to genomic sequencing data such as RNA sequencing (RNA-seq) and Global run-on sequencing (GRO-seq) and further improved for better resolution of sparse microbiome profiles^49^. The generalized fold change was calculated as the averaged difference between predefined quantiles (ranging from 0.1 to 0.9 in increments of 0.1 in this study) of the logarithmic control and adenoma, and between adenoma and cancer distributions.

### Model construction and features extraction

Following the differentially abundant ASVs analysis, we built RF models in the scikit-learn (V.0.19.2) package with stratified 10-fold cross-validation to distinguish adenoma from cancer or control. The features used for model building consist of patient metadata as well as differential ASVs and alpha diversity indices. The alpha diversity indices consisted of Shannon Index, Simpson Index, and Observed ASVs, while the patient metadata features consisted of age, sex, and BMI. The RF models were built with 501 estimator trees and each tree had 10% of the total features. And the stratified 10-fold cross-validation was used to configure training and testing data sets. Then an IFE step was used to optimize the performance of subsequent RF models. The top features from the top-performing model were selected as “important features” and the top microbial features as “biomarkers” (Supplementary Fig. 13). Finally, the AUC, accuracy, sensitivity, specificity, precision, and F1 score were used to evaluate the performance of the optimized models.

### Model evaluation

To assess the generalizability of microbial-based adenoma classifiers across contexts, such as geographic variation and technical differences in microbial data generation and processing over multiple patient populations, both study-to-study transfer validation and LODO validation were performed. In study-to-study transfer validation, classifiers were trained in one single study and externally assessed on all other studies (off-diagonal cells in Fig. 3a, b). Meanwhile, we applied a nested cross-validation procedure on the training study to calculate within-study accuracy (diagonal cells in Fig. 3a, b). In LODO validation, data from one study was set as the testing set, while data from the remaining three studies were pooled as the training set. We applied RF models in study-to-study transfer validation and LODO validation, the input features were the “important features”. Since multiple studies were involved, variations or batch effects are commonly observed^50^. To further improve the model’s ability to process batch effects among studies, fine-tuning model with bagging K-Nearest Neighbors (KNN) was performed in certain cases. KNN is measured by a distance metric of multiple features to reduce the dependence on the specific value of a feature, which can effectively avoid overfitting^51,52^.

To evaluate whether the important features would achieve the best performances in study-to-study transfer validation and LODO validation, we constructed models with three different sets of input features, including (1) all ASVs, (2) differential ASVs and (3) all important features. Then we sought to identify if there was a minimal set of important features that could achieve higher accuracy. A few of the top-ranking important features were always included in the minimal set as prior. We used the same methods as the study-to-study transfer validation and LODO validation and then calculated the average AUC of each testing study as each point in Fig. 3c, d. Finally, we compared the predictive values in the testing set across models with different sets of input features.

### Co-occurrence and clustering analysis

To construct co-occurrence networks of bacterial communities, network analysis was performed with the relative abundance of differential ASVs using the SparCC algorithm, which is known for its robustness for compositional data that are often characterized by diversity and sparsity of the members of the community^23^. Correlation coefficients were estimated as the average of 50 inference iterations with the default strength threshold. *P* values were calculated from 1000 bootstrap correlations. Correlation coefficients with *P* values < 0.05 (defined as significant) and with a magnitude above 0.1 (control versus adenoma) or above 0.3 (adenoma versus cancer) were selected for further visualization in Cytoscape (V.3.8.0). Modular structure and groups of highly interconnected nodes were analyzed using the MCODE application with standard parameters^25^.

To further analyze the co-occurrence of biomarkers, the relative abundances of biomarkers were discretized into binary values “positive” or “negative”. A sample was labeled “positive” when the relative abundance of biomarker ASV was above 0^14^. Based on the binarized markers-by-sample matrix, biomarkers were then clustered using the Jaccard index. Associations between clusters and metadata were calculated in a Cochran–Mantel–Haenszel test, using “study” as a blocking factor.

### The diagnostic ability of FIT for colorectal adenoma

To evaluate the diagnostic ability of traditional non-invasive test, FIT, we collected the publicly available FIT samples (including 172 control individuals and 198 adenoma patients) from a published study^27^. We constructed the RF models using important features, FIT or their combination for differentiating adenoma from control. The parameters of the RF models were the same as described in “Model construction and features extraction” section.

### Additional validation with independent studies and non-CRC diseases

As an external test, we used additional independent data to validate the performance of the important features to differentiate adenoma from cancer or control. Since the sequencing data of independent cohorts were not targeting the V4 region (details in Supplementary Table 2), ASVs from this dataset do not match with those of the discovery dataset. Consequently, we reconstructed RF models with the same hyperparameters as the discovery RF models. Considering the limited resolution of the 16S rRNA gene and incomplete reference database^53^, not all ASVs could be assigned at the species level. Thus all ASVs with the same taxonomy assignments (at genus level), as well as patient metadata (only used ASVs for validation cohort2 for lack of the patient metadata), were used as the input features.

To assess the specificity of the important features for colorectal adenoma, we examined the performances of these features in five non-CRC diseases (CD, UC, IBS, NAFLD, T2D)^43–46^. For each disease, RF models were constructed to discriminate the non-CRC diseases from controls. Similar to the validation with independent studies above, the input features were the ASVs with the same taxonomy assignments (at genus level) as the input features as well as patient metadata (only used ASVs, age, and sex as input features for CD and UC samples as BMI is not available) (Supplementary Data 15).

### Functional profile analysis

The functions of the gut microbiome were inferred from 16S rRNA sequences with PICRUSt2 (V.2.0.3-b) as previously published^54^. Functional profiles that have more than 80% samples with relative abundance < 1 × 10^−5^ and show up in less than three of the studies were removed. The differential analysis and generalized fold change calculations were performed on pathway profiles in the same way as on ASVs profiles (see Methods data preprocessing). Then, we evaluated the contribution of each ASV to overall differential pathways. The contribution was defined as the ratio of one ASV functional abundance to the total functional abundance of all ASVs in a given pathway.

### qRT-PCR validation

To quantify the abundance and expression of genes from two selected biosynthesis, qRT-PCR analysis was performed in triplicates on 7 healthy controls, 6 adenoma, and 30 CRC samples. For these samples, the gDNA was extracted with the FecalGen DNA Kit (Cat# e9604) according to the manufacturer’s instructions. We used the primes in Supplementary Table 6 for candidate genes; standard primers F515 and R806 for 16S rRNA. To perform the qRT-PCR reaction, the final primer concentration was diluted to 0.5 μM including 5 ng of gDNA in a 20 μl final reaction volume with the SYBR Green qPCR Mix (Thermo Fisher Scientific). The adopted qRT-PCR program was as follows: pre-denaturation at 95 °C for 10 min; denaturation at 95 °C for 15 s for 40 cycles; annealing at 60 °C for 60 s followed by melt curve analysis^14^. The qRT-PCR analysis was to calculate 2^−ΔΔCt^ values between candidate genes and 16S Ct values. The significance of the comparison between adenoma and control or CRC samples was tested by a two-sided Wilcoxon rank-sum test (*P* < 0.05).

### Reporting summary

Further information on research design is available in the Nature Research Reporting Summary linked to this article.

### Supplementary information

Supplementary Information

Descriptions of Additional Supplementary Files

Supplementary Data 1

Supplementary Data 2

Supplementary Data 3

Supplementary Data 4

Supplementary Data 5

Supplementary Data 6

Supplementary Data 7

Supplementary Data 8

Supplementary Data 9

Supplementary Data 10

Supplementary Data 11

Supplementary Data 12

Supplementary Data 13

Supplementary Data 14

Supplementary Data 15

Reporting Summary

### Source data

Source Data

## Data Availability

The raw 16S rRNA gene sequencing data are available from the Sequence Read Archive (SRA) (https://www.ncbi.nlm.nih.gov/sra) and European Nucleotide Archive (ENA) (https://www.ncbi.nlm.nih.gov/), with project ID: PRJNA389927, PRJEB6070, PRJNA290926, PRJNA362366, PRJNA534511, PRJNA280026, PRJEB28350, PRJNA544721, PRJNA541332, and PRJNA82111. The remaining data are available within the Article, Supplementary Information, or available from the authors upon request. Source data are provided with this paper.

## Code availability

The codes and scripts are available at https://github.com/Yuanqiwu/CRC (10.5281/zenodo.4739990)^55^. The customized code was written in Python 3.7.1 and R 3.5.2.
