# Supplementary material for: Identification of microbial markers across populations in early detection of colorectal cancer
Source: Nat Commun. 2021 May 24;12:3063. doi: 10.1038/s41467-021-23265-y (PMC8144394; doi:10.1038/s41467-021-23265-y)
Supplement: Supplementary file 1 — Supplementary Information [file 41467_2021_23265_MOESM1_ESM.pdf]

Identification of microbial markers across populations in early detection of colorectal cancer

Yuanqi Wu, Na Jiao, Ruixin Zhu, Yida Zhang, Dingfeng Wu, An-Jun Wang, Sa Fang, Liwen Tao, Yichen Li, Sijing Cheng, Xiaosheng He, Ping Lan, Chuan Tian, Ning-Ning Liu, Lixin Zhu

Supplementary Information

This file contains Supplementary Figures 1–17 and Supplementary Tables 1–6.

Table of Contents

Supplementary Figures 2

Supplementary Tables 19

Supplementary Figures

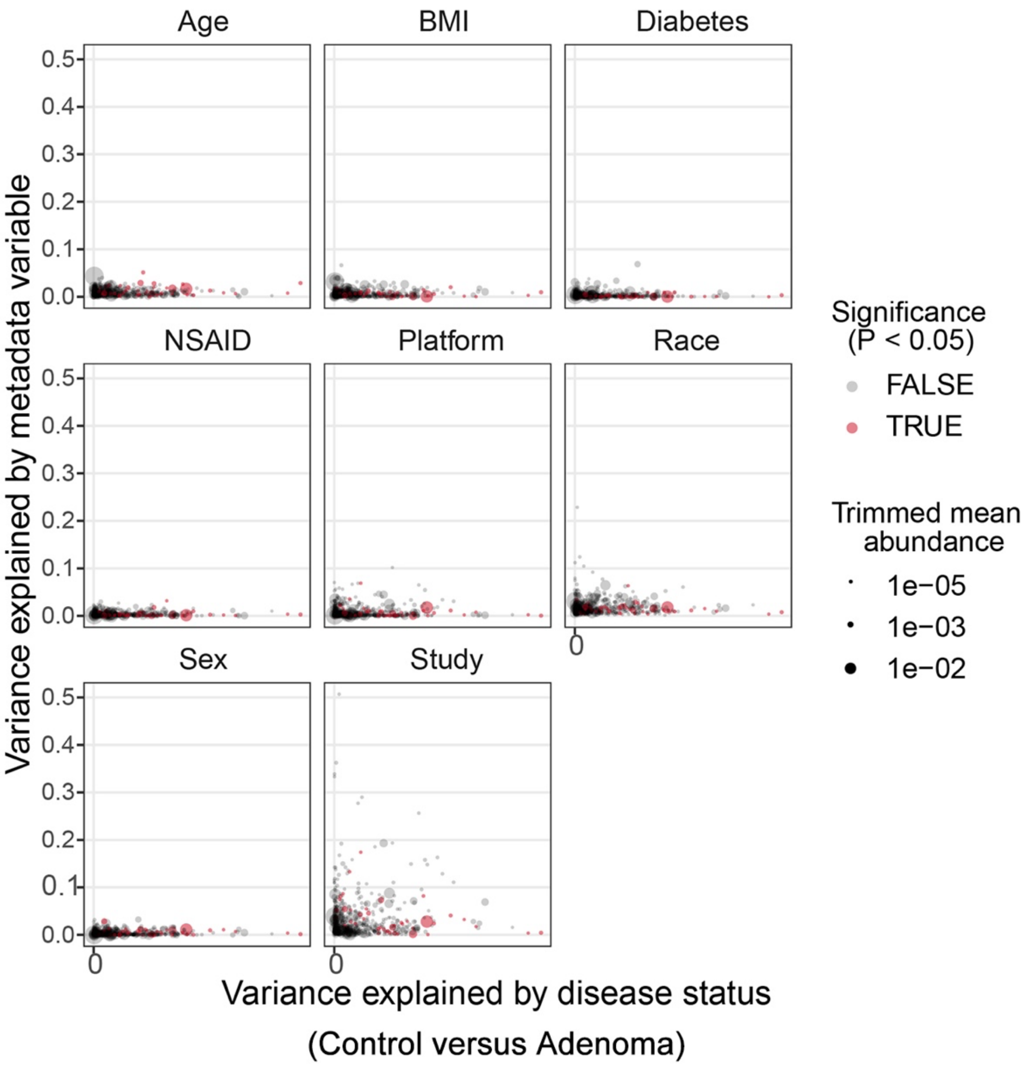

Supplementary Fig. 1 | Variance explained by putative confounding factors and disease status (Control versus Adenoma)

Variance explained by disease status (control versus adenoma; control, n=252; adenoma, n=306) is plotted against variance explained by different potential confounders (age, BMI, diabetes, NSAID, platform, race, sex and study) for individual ASVs. The abundance of each ASV is represented by the size of dot; the differentially abundant ASVs identified in the meta-analysis are highlighted in red. The variance explained by disease status was computed with the entire data. *P* values comparing between control and adenoma were from two-sided blocked Wilcoxon rank-sum tests (see Methods). Source data and exact *P* values are provided as a Source Data file.

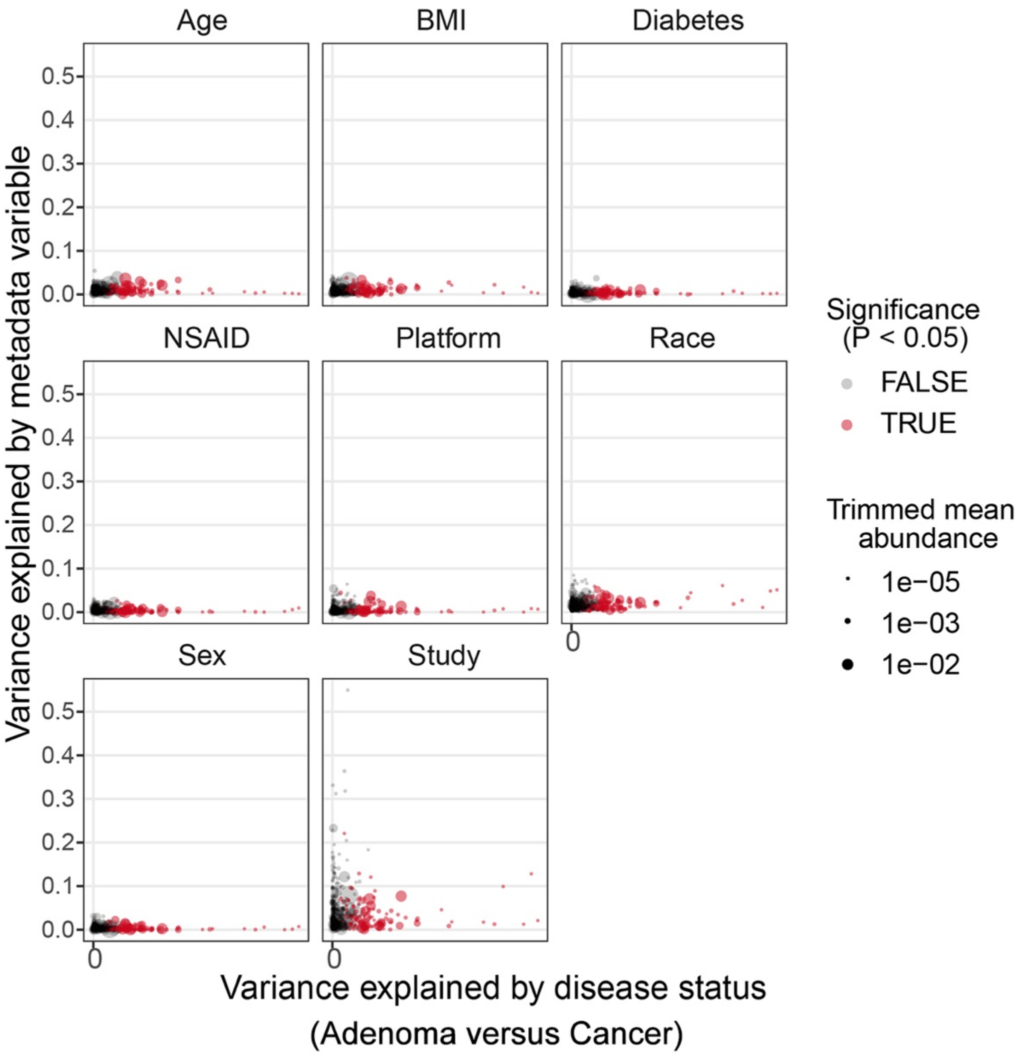

Supplementary Fig. 2 | Variance explained by putative confounding factors and disease status (Adenoma versus Cancer)

Variance explained by disease status (adenoma versus cancer; adenoma, n=306; cancer, n=217) is plotted against variance explained by different potential confounders (age, BMI, diabetes, NSAID, platform, race, sex and study) for individual ASVs. The abundance of each ASV is represented by the size of dot; the differentially abundant ASVs identified in the meta-analysis are highlighted in red. The variance explained by disease status was computed with the entire data. *P* values comparing between adenoma and cancer were from two-sided blocked Wilcoxon rank-sum tests (see Methods). Source data and exact *P* values are provided as a Source Data file.

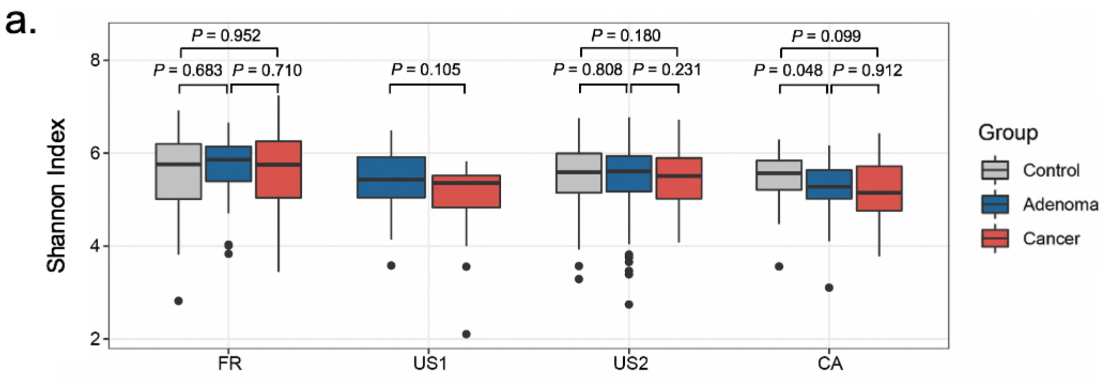

Group	*P*
Control-vs-Adenoma	0.600
Adenoma-vs-Cancer	0.074
Control-vs-Cancer	0.112

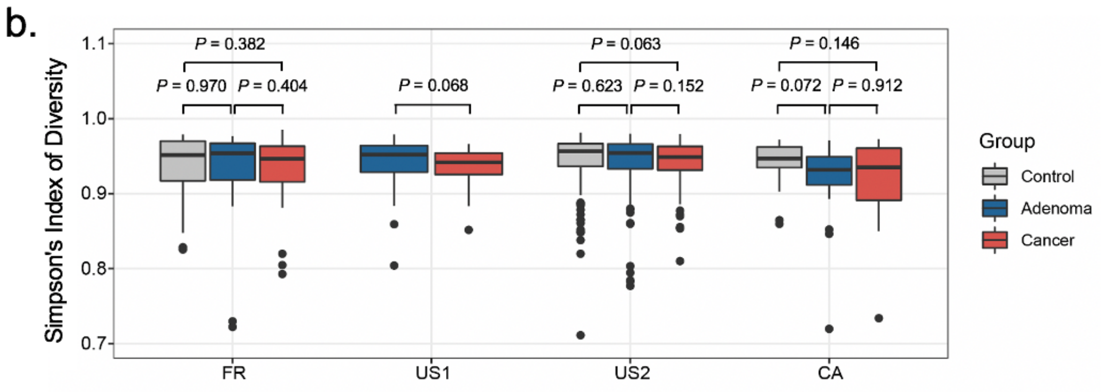

Group	*P*
Control-vs-Adenoma	0.365
Adenoma-vs-Cancer	0.043
Control-vs-Cancer	0.020

Supplementary Fig. 3 | Comparisons of alpha-diversity between different groups

Alpha diversity as measured with the (a) Shannon Index and (b) Simpson's Index of Diversity (defined as 1-$sump_i^2$) was computed with all ASVs in all samples (control, n=252; adenoma, n=306; cancer, n=217). *P* values of pairwise comparisons between two groups in each dataset (on top) were computed using a two-sided Wilcoxon rank-sum test. The combined *P* values (on the bottom table) were calculated using a two-sided blocked Wilcoxon rank-sum test by blocking "study". All boxplots represent the 25th–75th percentile of the distribution; the median is shown as a thick line in the box; the whiskers extend up to the most extreme points within 1.5-fold IQR, and outliers are represented as dots. Source data are provided as a Source Data file.

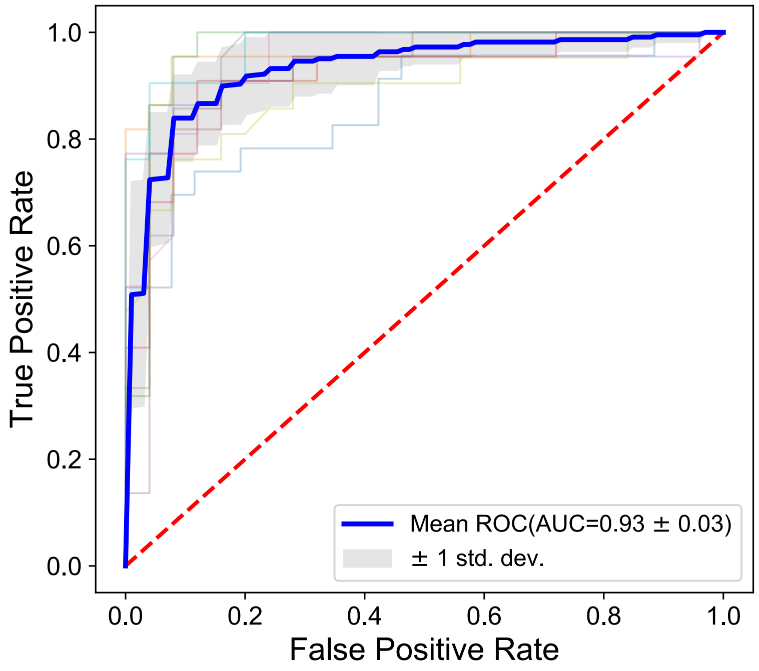

Supplementary Fig. 4 | Performance of the RF Models for CRC detection

Receiver operating characteristic (ROC) curve of the RF model (control versus cancer) constructed using the relative abundances of the 35 ASVs together with age and BMI.

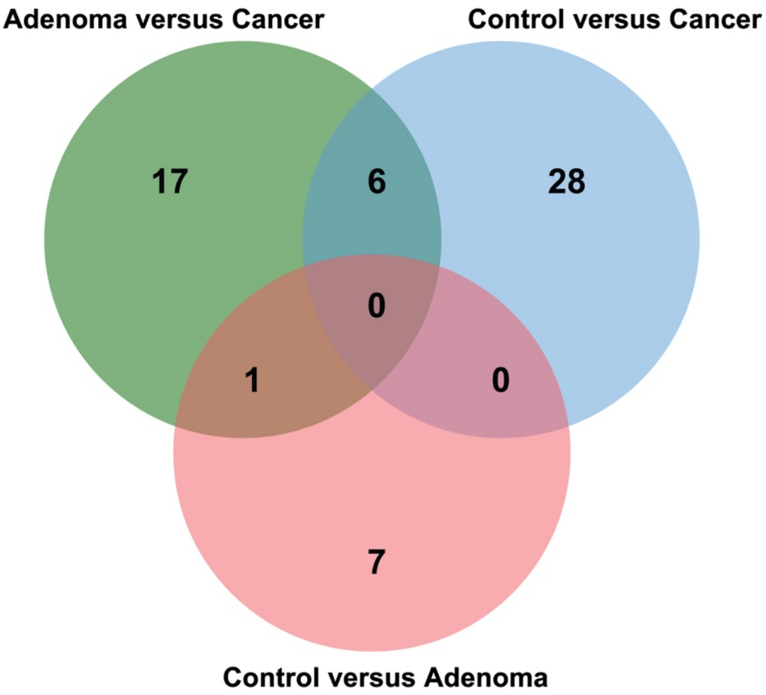

Supplementary Fig. 5 | Overlap of three sets of biomarkers in Venn diagram

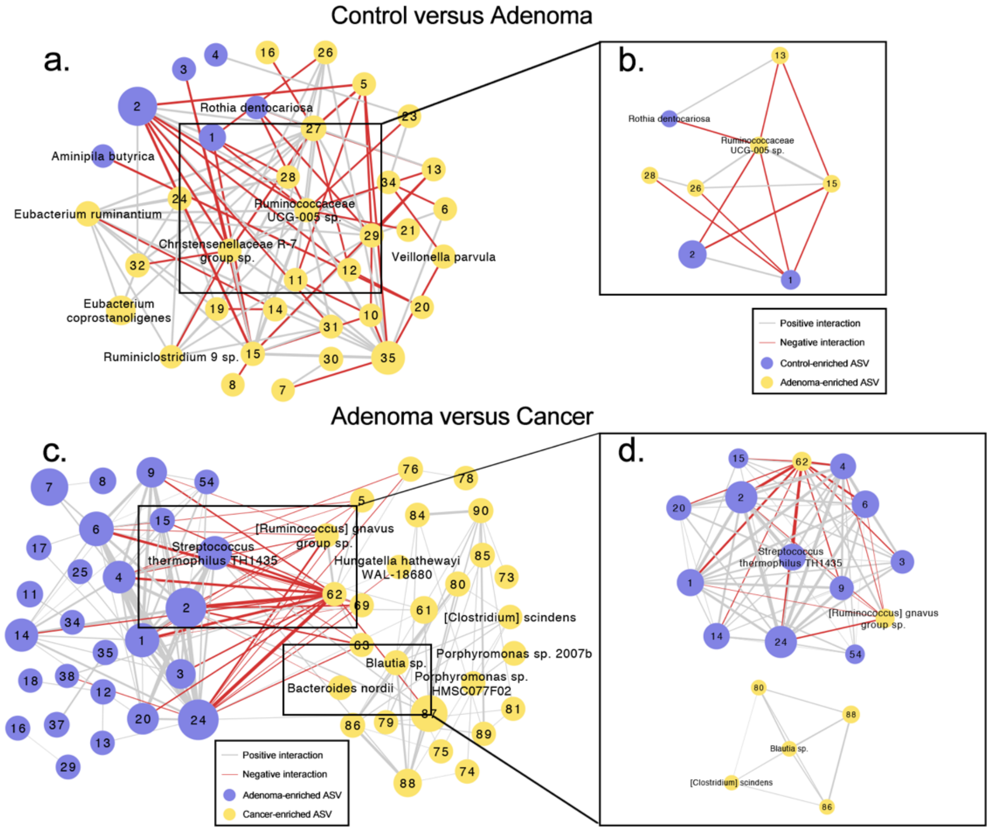

Supplementary Fig. 6 | Microbial correlation networks for biomarkers. (a) Correlation network of differential ASVs between adenoma and control (n=43 differential ASVs) and (c) Correlation network of differential ASVs between adenoma and CRC (n=117 differential ASVs). Correlation coefficients were calculated by the SparCC algorithm. Modules (b) and (d) were constructed using the MCODE application from (a) and (c), respectively. Node size represents mean ASV abundance; biomarker ASVs are annotated to species; other differential ASVs are denoted by node numbers; Edges indicate correlations: the edge thickness represents the magnitude and the color represents the sign of the correlation (gray, positive; red, negative).

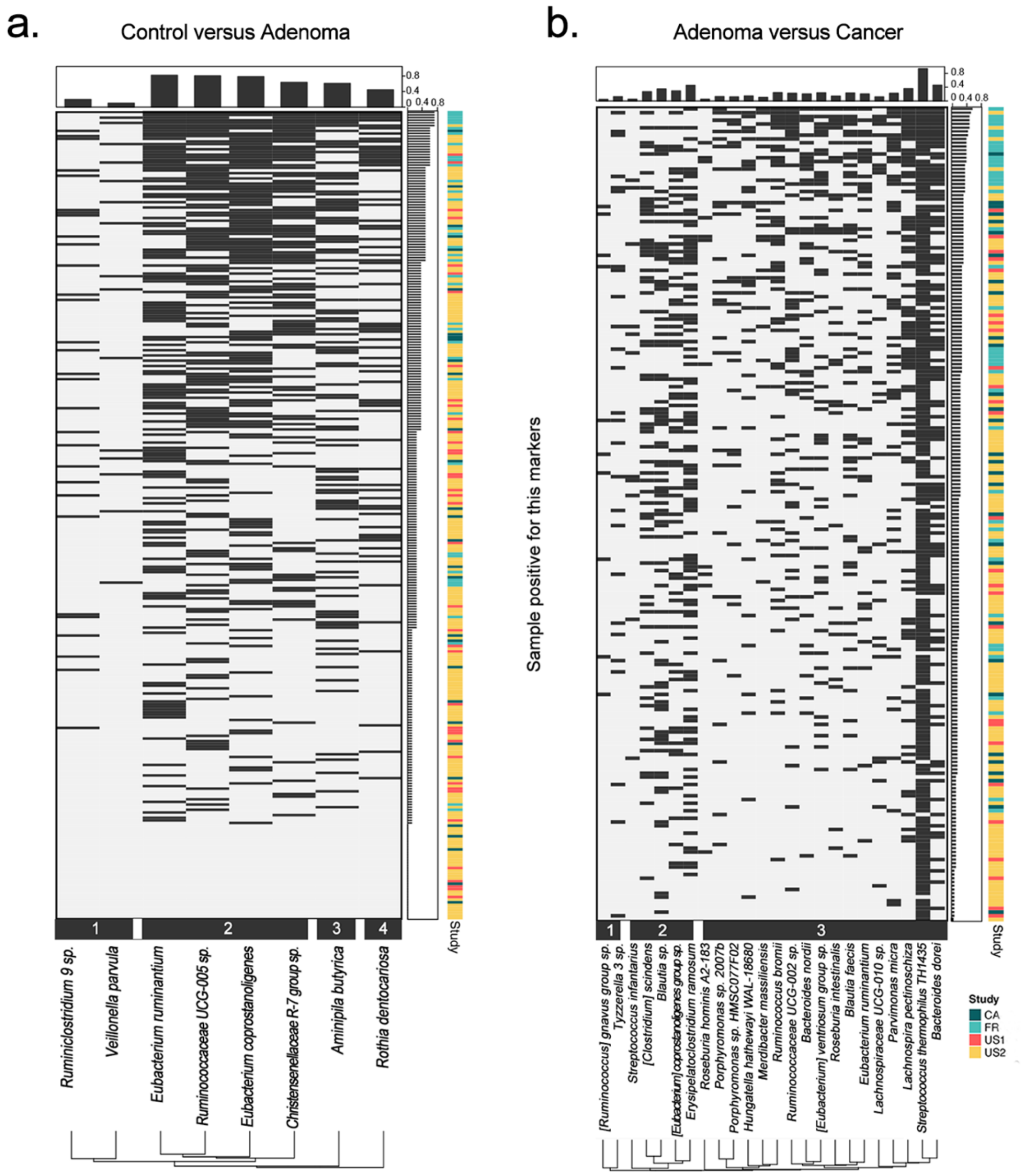

Supplementary Fig. 7 | Co-occurrence analysis of biomarkers for distinguishing adenoma from control or CRC

For all patients with (a) adenoma (n=306) or (b) CRC (n=217), the heatmaps show whether the respective sample is positive for each of the biomarkers. Samples were ordered by the sum of positive biomarkers, and the biomarkers were grouped into four clusters (a) or three clusters (b) based on the Jaccard index of positive samples. Source data are provided as a Source Data file.

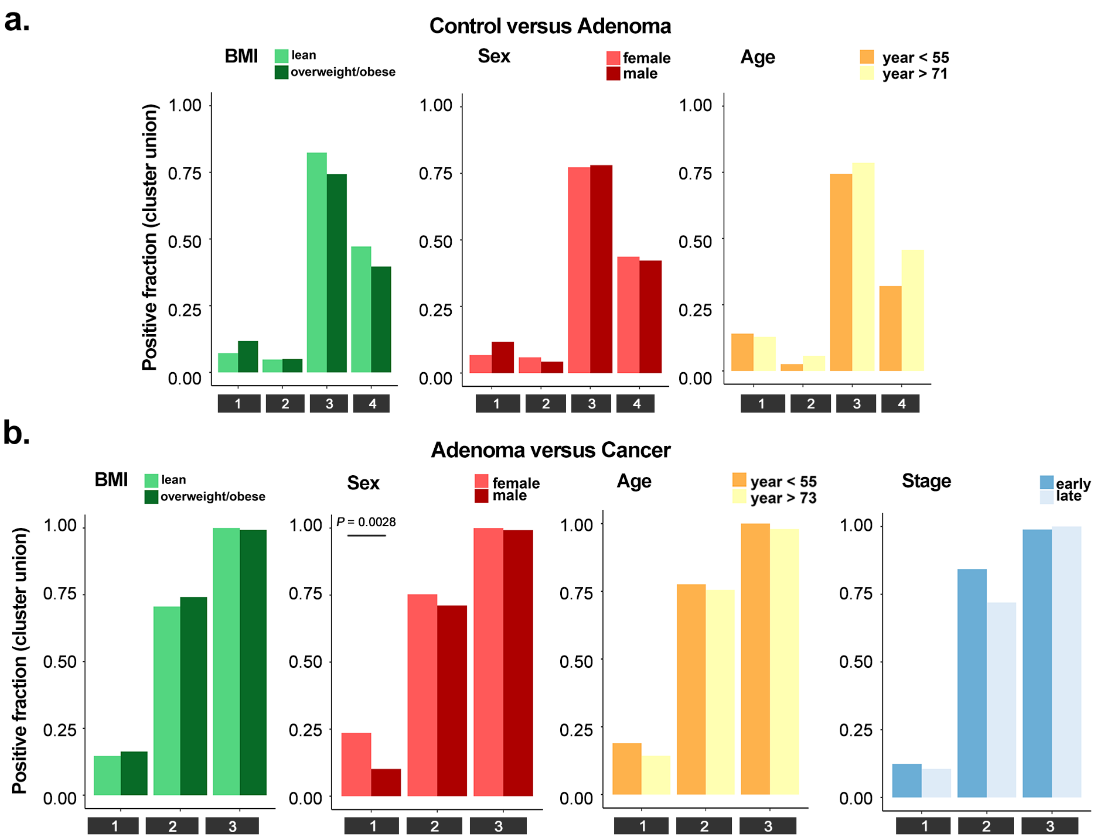

Supplementary Fig. 8 | Co-occurrence of biomarkers identified clusters linked to different patient characteristics

The barplots manifested the positive fractions for clusters of biomarkers between adenoma and control (n=8 biomarkers) (a), or between adenoma and CRC (n=24 biomarkers) (b) broken down by patient subgroups based on sex (a-b), age (a-b), BMI (a-b) and stage (b), respectively. The significant associations between adenoma subgroups (a) or CRC subgroups (b) and biomarker clusters were identified by the Cochran–Mantel–Haenszel test blocked for “study”. Source data are provided as a Source Data file.

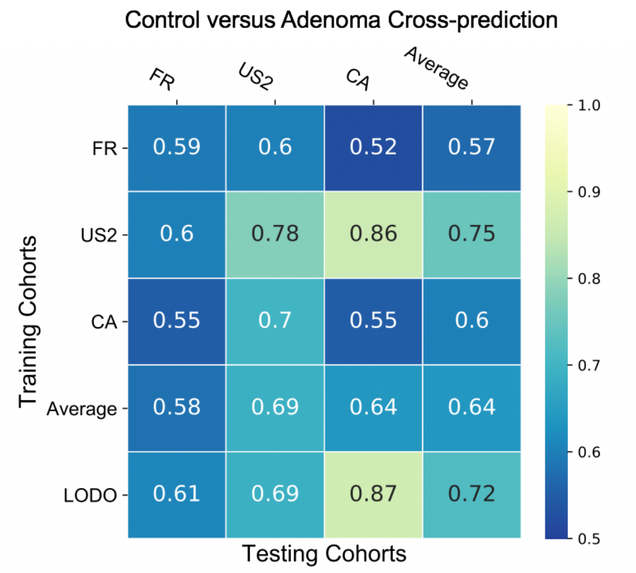

Supplementary Fig. 9 | The study-to-study and LODO validations for differentiating adenoma from control using RF classifiers

Values on the diagonal refer to the results of cross-validation within each study; Off-diagonal values refer to the AUC values obtained from cross-cohort validations, which train the classifier on the study of the corresponding row and apply it to the study of the corresponding column; The LODO values refer to the performances obtained by training the classifier using all but the study of the corresponding column and apply it to the study of the corresponding column (see Methods).

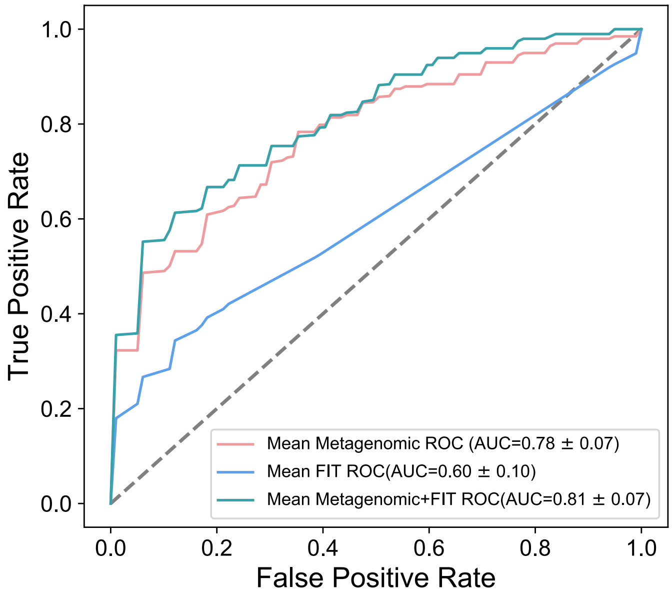

Supplementary Fig. 10 | Improved adenoma diagnostic ability by combining important features with FIT tests

AUC values for the prediction of colorectal adenoma using selected important features, FIT or a combination of both were indicated. AUC value was highest from the combination test.

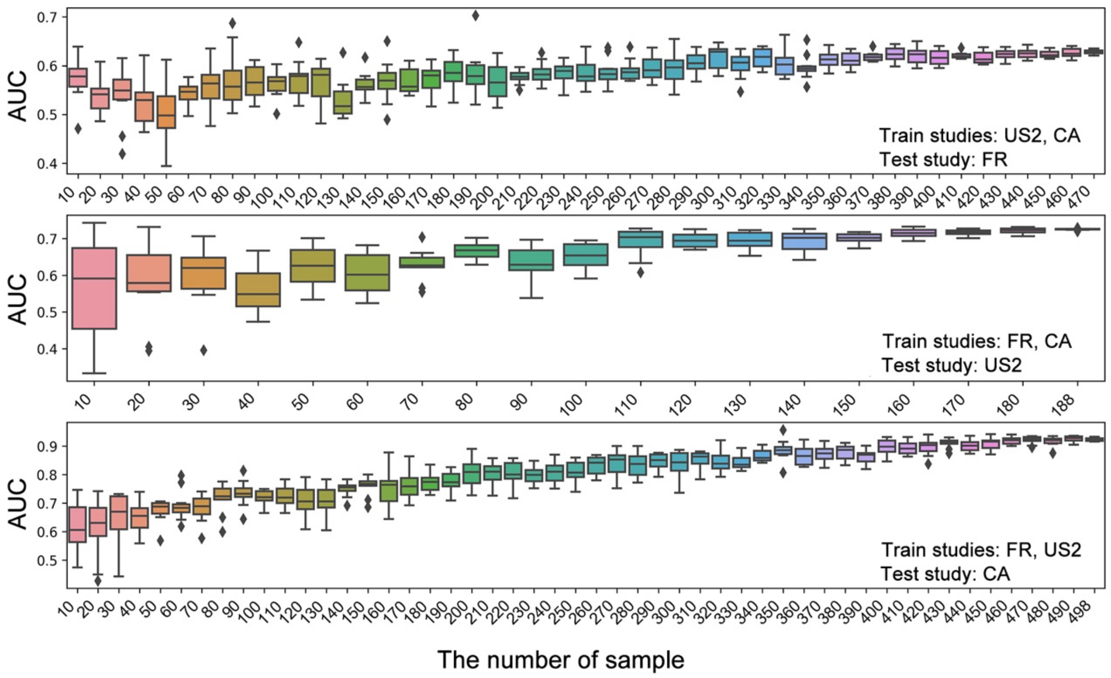

Supplementary Fig. 11 | LODO validations at increasing numbers of the training samples. With control (n=252) versus adenoma (n=306) bagging KNN classifiers, the AUC values of LODO validations increased when adding training samples. All boxplots represent 25th–75th percentile of the distribution; the median is shown in thick line at the middle of the box; the whiskers extend up to values within 1.5 times of IQR, and outliers are represented by dots. Source data are provided as a Source Data file.

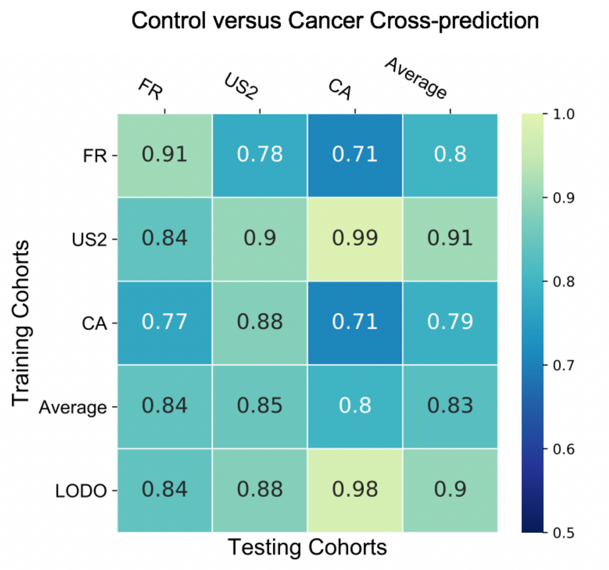

Supplementary Fig. 12 | The study-to-study and LODO validation for differentiating control from CRC using RF classifiers

Values on the diagonal refer to the results of cross-validation within each study; Off-diagonal values refer to the AUC values obtained from cross-cohort validations, which train the classifier on the study of the corresponding row and apply it to the study of the corresponding column; The LODO values refer to the performances obtained by training the classifier using all but the study of the corresponding column and apply it to the study of the corresponding column (see Methods).

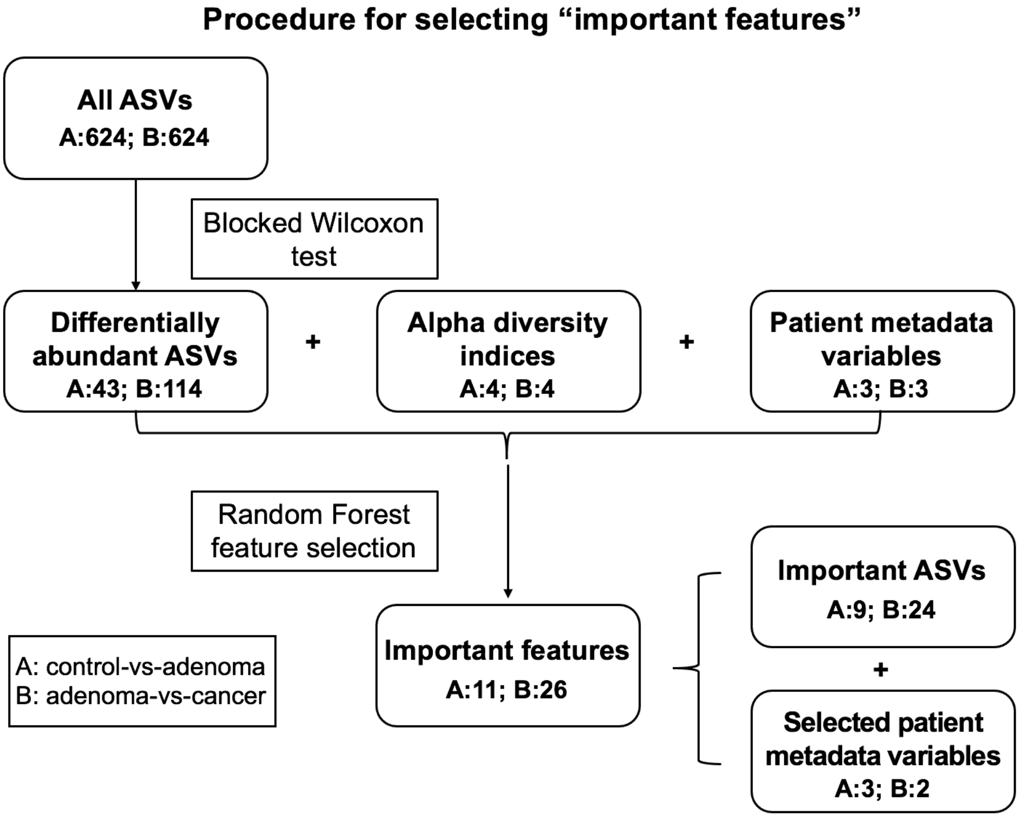

Supplementary Fig. 13 | The procedure for selecting “important features”. The differentially abundant ASVs were identified using a two-sided blocked Wilcoxon rank-sum test applied on all ASVs. Besides using differential ASVs as key metrics, alpha diversity indices including Shannon Index, Simpson Index and Observed ASVs, and three patient metadata variables, age, sex and BMI were also included in the Random Forest model building. The important ASVs and selected patient metadata variables were included in important features. The number of ASVs/variables is shown in each step. A: control-vs-adenoma model; B: adenoma-vs-cancer model.

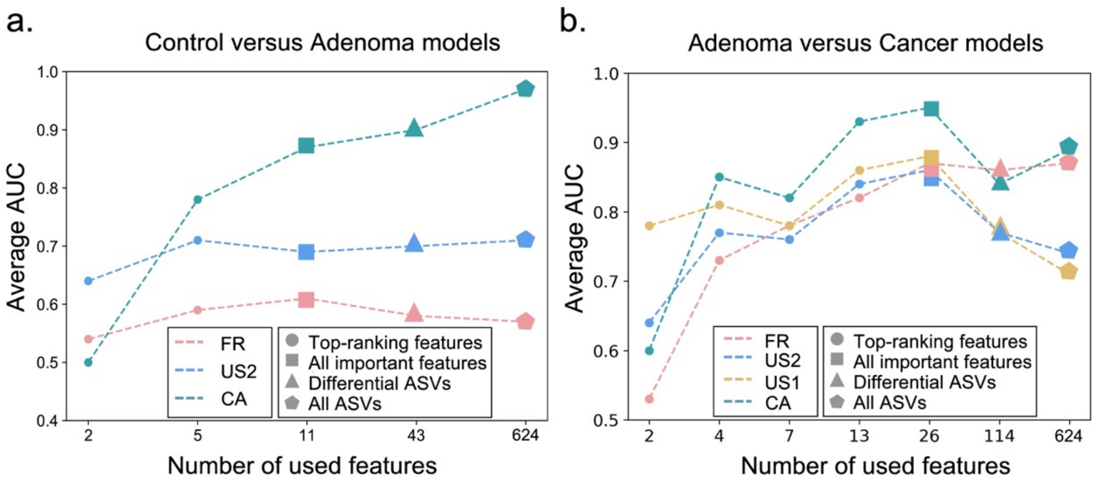

Supplementary Fig. 14 | Prediction performances of LODO validation classifiers with different sets of features

Average AUC of LODO validation classifiers for control versus adenoma (a) and adenoma versus cancer (b) with different sets of features. Shapes represent different sets of input features. The x-axis indicates different numbers of features. Colors represent different studies. Source data are provided as a Source Data file.

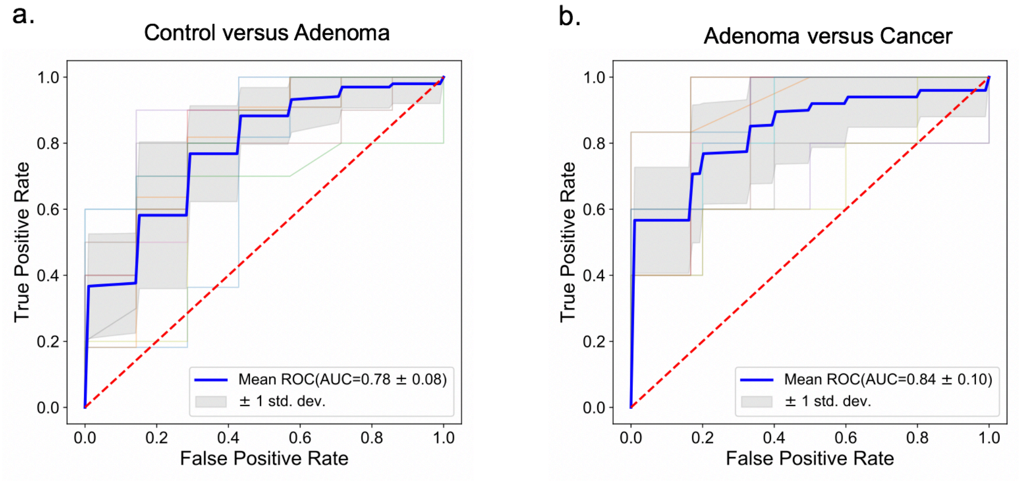

	AUC	Accuracy	Sensitivity	Specificity	Precisions	F1 score
Control-vs-Adenoma	0.78±0.08	0.70±0.10	0.76±0.14	0.59±0.17	0.71±0.07	0.77±0.08
Adenoma-vs-Cancer	0.84±0.10	0.79±0.08	0.79±0.14	0.80±0.15	0.78±0.09	0.72±0.06

Supplementary Fig. 15 | Validation performance of two independent cohorts for discriminating adenoma from control (a) and CRC (b). Metrices of models are shown in the bottom table and data are presented with average ± s.d..

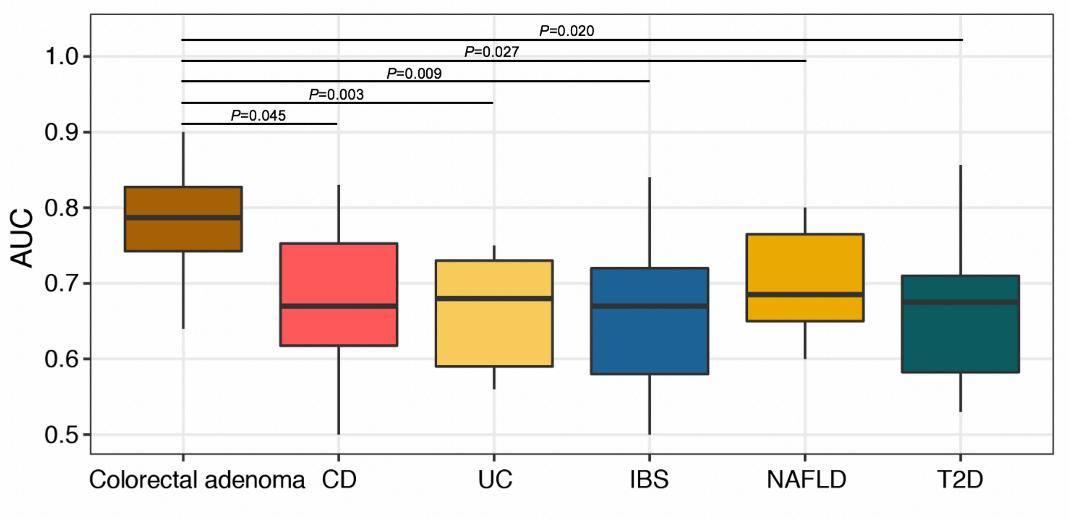

Supplementary Fig. 16 | The specificity of the adenoma prediction model. The comparison of the performances of important features among different microbiome-linked disease models: adenoma (n=102) versus control (n=70) model, CD (n=61) versus control (n=18) model, UC (n=47) versus control (n=18) model, IBS (n=84) versus control (n=44) model, NAFLD (n=18) versus control (n=51) model, and T2D (n=48) versus control (n=214) model. All boxplots represent the 25th–75th percentile of the distribution; the median is shown in thick line at the middle of the box; the whiskers extending up to the most extreme points within 1.5-fold IQR. *P* values were calculated with a two-sided Wilcoxon rank-sum test. Source data are provided as a Source Data file.

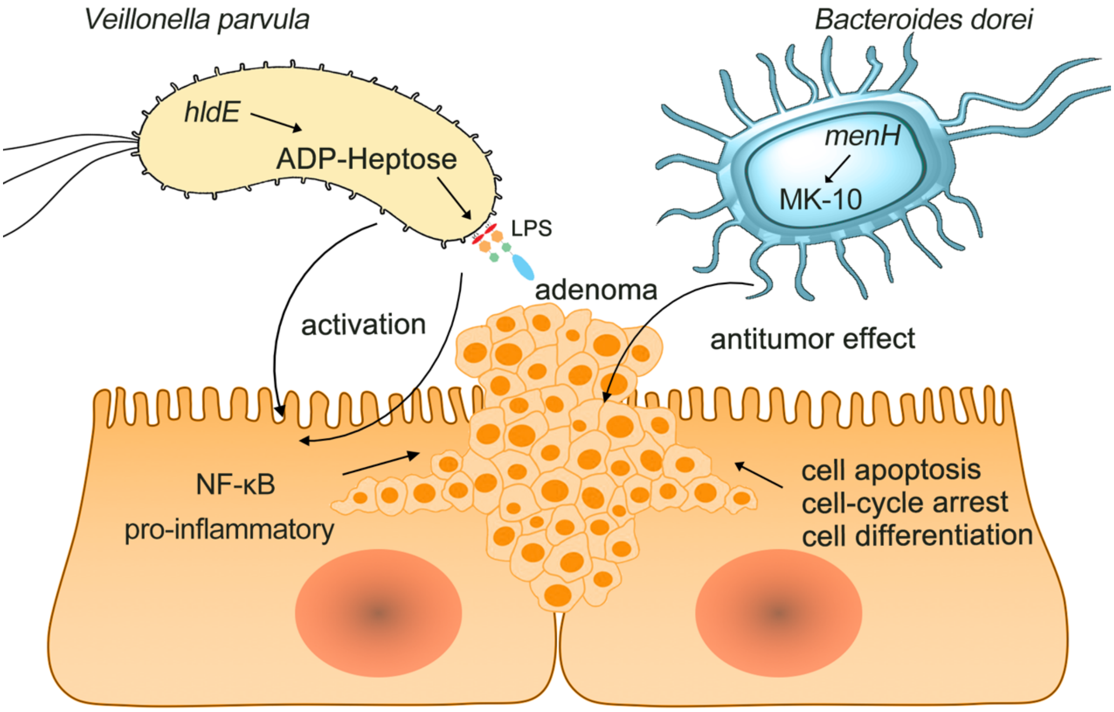

Supplementary Fig. 17 | Potential mechanisms for microbial markers participating in the pathogenesis of colorectal adenoma and cancer

The biosynthesis of ADP-heptose coded by *hldE* and etc genes is associated with the activation of NF-κB and consequently a strong inflammatory response, while the MK-10 pathway coded by *menH* and etc genes plays an antitumor role via regulations of cell-cycle arrest, cell differentiation and cell apoptosis.

Supplementary Tables

Supplementary Table 1 | Metrics of control versus adenoma and adenoma versus cancer model performances

	AUC	Accuracy	Sensitivity	Specificity	Precisions	F1 score
Control-vs-Adenoma[#]	0.80±0.07[*]	0.73±0.06	0.82±0.08	0.62±0.12	0.73±0.06	0.77±0.05
Adenoma-vs-Cancer[$]	0.89±0.03	0.80±0.03	0.66±0.11	0.90±0.03	0.83±0.04	0.72±0.06

*: Data are presented as average ± s.d., calculated from the results of stratified 10-fold cross-validation.

[#]: The Control-vs-Adenoma model was constructed with control (n=252) and adenoma (n=306) samples.

[$]: The Adenoma-vs-Cancer model was constructed with adenoma (n=306) and cancer (n=217) samples.

Supplementary Table 2 | Characteristics of the independent cohorts and non-CRC disease studies

Study	Group (N*)	Age (average±s.d. #)	BMI (average±s.d. #)	Sex F(%)/M(%)†	No. of reads (average±s.d. #)	Country
Validation cohort1	control(70)	63.12±8.05	27.41±5.52	36.27/63.73	19,149±15,910	USA
	adenoma(102)	61.46±9.03	26.81±4.41	40.00/60.00		
Validation cohort2	adenoma(57)	NA	NA	NA	11,079±5,552	China
	cancer(52)					
NAFLD$	control(51)	45.85±19.86	26.07±6.83	70.59/29.41	34,517±14,495	USA
	case(18)	54.00±14.86	31.08±6.65	66.67/33.33		
IBS$	control(44)	39.05±12.92	23.82±3.72	43.18/56.82	21,920±4,638	China
	case(84)	42.01±11.96	23.47±3.52	34.52/65.48		
T2D$	control(214)	36.34±13.99	26.38±5.47	78.50/21.5	68,245±33,780	USA
	case(48)	51.44±9.26	32.47±6.95	64.58/35.42		
IBD$	control(18)	25±2.74	NA	33.33/66.67	4,231±575	USA
	CD case(61)	33.51±19.76		36.07/63.93		
	UC case(47)	41.81±18.41		48.94/51.06		

* number of samples;

# standard deviation;

† the ratio of percentage of female and male;

$ the accession numbers of NAFLD, IBS, T2D and IBD studies were PRJEB28350, PRJNA544721, PRJNA541332 and PRJNA82111;

Note: validation cohort 1: V1-V4; validation cohort 2: V3-V4; NAFLD: V4 single; IBS: V3-V4 single; T2D: V4; CD and UC: V3-V5.

Supplementary Table 3 | Altered abundances for the microbial genes involved in ADP-heptose biosynthesis

ADP-heptose biosynthesis (Control versus Adenoma)			
Enzyme	GFOLD-meta$	pvalue-meta#	Gene name
EC: 5.3.1.28	0.0712	0.0046	*gmhA*
EC: 2.7.1.167/ EC: 2.7.7.70*	0.0838	0.0042	*hldE*
EC: 3.1.3.82	0.0706	0.0014	*gmhB*
EC: 5.1.3.20	0.0675	0.0185	*rfaD*

$ Mean of generalized fold changes across studies, GFOLD-meta >0: gene enriched in adenoma compared with control; <0: gene enriched in control compared with adenoma;

# Meta-analysis *P*-value calculated by two-sided blocked Wilcoxon rank-sum test;

* EC: 2.7.1.167 and EC: 2.7.7.70 have the same gene name.

Supplementary Table 4 | Altered abundances for the microbial genes involved in MK-10 biosynthesis

MK-10 biosynthesis (Adenoma versus Cancer)			
Enzyme	GFOLD-meta†	pvalue-meta#	Gene name
EC: 4.2.1.113	0.0798	0.0259	*menC*
EC: 4.2.99.20	0.0870	0.0459	*menH*
EC: 5.4.4.2	0.0967	0.0491	*menF*

† Mean of generalized fold changes across studies, GFOLD-meta >0: gene enriched in cancer compared with adenoma; <0: gene enriched in adenoma compared with cancer;

# Meta-analysis *P*-value calculated by two-sided blocked Wilcoxon rank-sum test.

Supplementary Table 5 | Characteristics of human samples for qRT-PCR

	Control (n=7)	Adenoma (n=6)	CRC(n=30)
Sex (F/M)	4/3	2/4	12/18
Age (years)	48-71	50-70	41-77
BMI(average ± s.d. #)	24.07±0.65	21.95±2.2.0	22.74±1.95

standard deviation.

Supplementary Table 6 | Primers for qRT-PCR analysis of the microbial genes

ID	Primer name	Primer sequence (5'to3')
1	fNL303-forward *GmhA*	TCTCCCGCTATGTTGAAGCG
2	rNL304-reverse *GmhA*	TCAATATCCGCCGTACCAGC
3	fNL305-forward *hIdE*	TCGTCGTATGGCGGTATTGG
4	rNL306-reverse *hIdE*	GCAGCAATCACTTCAGCACC
5	fNL307-forward *gmhB*	ACATCCGGGGATGCTTTTGT
6	rNL308-reverse *gmhB*	CCAGCACTTTTGTTCCCACG
7	fNL311-forward *rfaD*	AGCGTCGCTTTCCATCTCAA
8	rNL312-reverse *rfaD*	CCGAGATTGAAGATGCCGGA
9	fNL313-forward *menC*	GGCGGTGATCAGTTCTTCCA
10	rNL314-reverse *menC*	CATCAGATCCAGCGTGTCCA
11	fNL317-forward *menH*	GTTGATCTCCCAGGTCACGG
12	rNL318-reverse *menH*	TGTTCAGCATTTTGCAGCCC
13	fNL319-forward *menF*	ATCTTCGCCGCTGTATCTGG
14	rNL320-reverse *menF*	AATTTTTGCTGAGCGCAGGG
