# Supplementary material for: Identification of microbial markers across populations in early detection of colorectal cancer
Source: Nat Commun. 2021 May 24;12:3063. doi: 10.1038/s41467-021-23265-y (PMC8144394; doi:10.1038/s41467-021-23265-y)
Supplement: Supplementary file 2 — Descriptions of Additional Supplementary Files [file 41467_2021_23265_MOESM2_ESM.pdf]

Descriptions of Additional Supplementary Files

Supplementary Data 1
Description: Bacterial phyla differential tests (Pvalues) in healthy controls, adenomas and CRC

Supplementary Data 2
Description: Differentially abundant ASVs between control and adenoma (P<0.05).

Supplementary Data 3
Description: Differentially abundant ASVs between adenoma and cancer (P<0.05).

Supplementary Data 4
Description: Gut microbiome-based markers discriminated between control and adenoma.

Supplementary Data 5
Description: Gut microbiome-based markers discriminated between adenoma and cancer.

Supplementary Data 6
Description: Biomarkers of two meta-analyses and this study for distinguishing control and CRC.

Supplementary Data 7
Description: Node numbers of differential ASVs between control and adenoma.

Supplementary Data 8
Description: Node numbers of differential ASVs between adenoma and cancer.

Supplementary Data 9
Description: The input features of RF model for validation cohort 1.

Supplementary Data 10
Description: The input features of RF model for validation cohort 2.

Supplementary Data 11
Description: Differentially abundant pathways between control and adenoma (P<0.05)

Supplementary Data 12
Description: Differentially abundant pathways between adenoma and cancer (P<0.05).

Supplementary Data 13
Description: Average distribution of each ASV of healthy subjects and adenoma patients.

Supplementary Data 14
Description: Average distribution of each ASV of adenoma patients and CRC.

Supplementary Data 15
Description: The input feature of RF models for non-CRC diseases.
