# Supplementary material for: Identification of microbial markers across populations in early detection of colorectal cancer
Source: Nat Commun. 2021 May 24;12:3063. doi: 10.1038/s41467-021-23265-y (PMC8144394; doi:10.1038/s41467-021-23265-y)
Supplement: Supplementary file 18 — Reporting Summary [file 41467_2021_23265_MOESM18_ESM.pdf]

nature research

Corresponding author(s): Ruixin Zhu, Chuan Tian, Ning-Ning Liu, Lixin Zhu

Last updated by author(s): Apr 02, 2021

Reporting Summary

Nature Research wishes to improve the reproducibility of the work that we publish. This form provides structure for consistency and transparency in reporting. For further information on Nature Research policies, see our Editorial Policies and the Editorial Policy Checklist.

Statistics

For all statistical analyses, confirm that the following items are present in the figure legend, table legend, main text, or Methods section.

n/a	Confirmed	
☐	☒	The exact sample size (*n*) for each experimental group/condition, given as a discrete number and unit of measurement
☐	☒	A statement on whether measurements were taken from distinct samples or whether the same sample was measured repeatedly
☐	☒	The statistical test(s) used AND whether they are one- or two-sided<br>*Only common tests should be described solely by name; describe more complex techniques in the Methods section.*
☐	☒	A description of all covariates tested
☐	☒	A description of any assumptions or corrections, such as tests of normality and adjustment for multiple comparisons
☐	☒	A full description of the statistical parameters including central tendency (e.g. means) or other basic estimates (e.g. regression coefficient) AND variation (e.g. standard deviation) or associated estimates of uncertainty (e.g. confidence intervals)
☐	☒	For null hypothesis testing, the test statistic (e.g. *F*, *t*, *r*) with confidence intervals, effect sizes, degrees of freedom and *P* value noted<br>*Give P values as exact values whenever suitable.*
☒	☐	For Bayesian analysis, information on the choice of priors and Markov chain Monte Carlo settings
☒	☐	For hierarchical and complex designs, identification of the appropriate level for tests and full reporting of outcomes
☐	☒	Estimates of effect sizes (e.g. Cohen's *d*, Pearson's *r*), indicating how they were calculated

Our web collection on statistics for biologists contains articles on many of the points above.

Software and code

Policy information about availability of computer code

Data collection	SRA toolkit (V.2.9.1) was used to download data.
Data analysis	The 16S rRNA sequencing data were analyzed using DADA2 (V.2018.11), Fast-Tree (V.2018.11), MAFFT (V.2018.11) plugin wrapped in Quantitative Insights Into Microbial Ecology (QIIME2 V.2018.11) .The functions of gut microbiome were inferred from 16S rRNA sequences with Picrust2-2.0.3-b. The models were constructed using scikit-learn (V.0.19.2). Network visualization was used by Cytoscape (V.3.8.0). The codes and scripts are available on https://github.com/Yuanqiwu/CRC. The custom code is written in Python 3.7.1 and R 3.5.2.

For manuscripts utilizing custom algorithms or software that are central to the research but not yet described in published literature, software must be made available to editors and reviewers. We strongly encourage code deposition in a community repository (e.g. GitHub). See the Nature Research guidelines for submitting code & software for further information.

Data

Policy information about availability of data

All manuscripts must include a data availability statement. This statement should provide the following information, where applicable:

- Accession codes, unique identifiers, or web links for publicly available datasets
- A list of figures that have associated raw data
- A description of any restrictions on data availability

The raw 16S rRNA gene sequencing data are available from the Sequence Read Archive (SRA) (https://www.ncbi.nlm.nih.gov/sra) and European Nucleotide Archive (ENA) (https://www.ncbi.nlm.nih.gov/), with project ID: PRJNA389927 [https://www.ncbi.nlm.nih.gov/bioproject/?term=PRJNA389927], PRJEB6070 [https://www.ncbi.nlm.nih.gov/bioproject/?term=PRJEB6070], PRJNA290926 [https://www.ncbi.nlm.nih.gov/bioproject/?term=PRJNA290926], PRJNA362366 [https://www.ncbi.nlm.nih.gov/bioproject/?term=PRJNA362366], PRJNA534511 [https://www.ncbi.nlm.nih.gov/bioproject/?term=PRJNA534511], PRJNA280026 [https://www.ncbi.nlm.nih.gov/bioproject/?term=PRJNA280026], PRJEB28350 [https://www.ncbi.nlm.nih.gov/bioproject/?term=PRJEB28350], PRJNA544721 [https://

www.ncbi.nlm.nih.gov/bioproject/?term=PRJNA544721], PRJNA541332 [https://www.ncbi.nlm.nih.gov/bioproject/?term=PRJNA541332] and PRJNA82111 [https://www.ncbi.nlm.nih.gov/bioproject/?term=PRJNA82111]. Source data are provided with this paper.

Field-specific reporting

Please select the one below that is the best fit for your research. If you are not sure, read the appropriate sections before making your selection.

[x] Life sciences [ ] Behavioural & social sciences [ ] Ecological, evolutionary & environmental sciences

For a reference copy of the document with all sections, see nature.com/documents/nr-reporting-summary-flat.pdf

Life sciences study design

All studies must disclose on these points even when the disclosure is negative.

Sample size	No sample size calculation was performed for this analysis, all publicly available data sets (see Methods, Table S1 and Supplementary Table 2) meeting a minimal set of inclusion criteria were included.
Data exclusions	A sample (SRR5184891 in PRJNA362366) sequenced less than 6,000 reads was excluded from the analysis.
Replication	All attempts at replicating qRT-PCR experiments were successful.
Randomization	Not applicable for this observational case-control analysis.
Blinding	Blinding was not possible because statistical analyses depended on information about cancer status (statistical tests for differences between groups and supervised statistical modeling).

Reporting for specific materials, systems and methods

We require information from authors about some types of materials, experimental systems and methods used in many studies. Here, indicate whether each material, system or method listed is relevant to your study. If you are not sure if a list item applies to your research, read the appropriate section before selecting a response.

Materials & experimental systems

n/a	Involved in the study	
[x]	[ ]	Antibodies
[x]	[ ]	Eukaryotic cell lines
[x]	[ ]	Palaeontology and archaeology
[x]	[ ]	Animals and other organisms
[ ]	[x]	Human research participants
[x]	[ ]	Clinical data
[x]	[ ]	Dual use research of concern

Methods

n/a	Involved in the study	
[x]	[ ]	ChIP-seq
[x]	[ ]	Flow cytometry
[x]	[ ]	MRI-based neuroimaging

Human research participants

Policy information about studies involving human research participants

Population characteristics	In our analysis there is heterogeneity in the population characteristics recorded by the primary studies. Participant age, sex and BMI were recorded for all subjects (see Supplementary Table 5).
Recruitment	This work includes heterogeneous recruitment procedures performed in primary studies. All the samples used in qRT-PCR analysis including the control, adenoma and cancer patients were collected from Fudan University Shanghai Tumor Center with the informed consent. For the patients' recruitment and sample collection, they were all approved by the Medical Ethics Committee of Fudan University Shanghai Tumor Center.
Ethics oversight	Patient recruitment and sample collection were approved by the Medical Ethics Committee of Fudan University Shanghai Tumor Center. Written informed consent was obtained from each participant. This study protocol is in agreement with the world medical association declaration of Helsinki (2008) and the Belmont Report.

Note that full information on the approval of the study protocol must also be provided in the manuscript.
